# Advances in Zn-MOF-Based Materials for Electrochemical and Fluorescence Sensing Applications

**DOI:** 10.3390/s26113511

**Published:** 2026-06-02

**Authors:** Khursheed Ahmad, Shanmugam Vignesh, Tae Hwan Oh

**Affiliations:** School of Chemical Engineering, Yeungnam University, 280 Daehak-Ro, Gyeongsan 38541, Republic of Korea; vigneshattur1@gmail.com

**Keywords:** Zn-MOF, sensors, biosensors, biomolecules, pollutants

## Abstract

Metal–organic frameworks (MOFs) exhibit high specific surface area and porosity, which may facilitate electron transfer during electrochemical reactions. Therefore, it is clear that MOFs are promising materials for the development of electrochemical sensors. In particular, zinc (Zn) based MOFs offer several advantages such as high specific surface area, porosity, environmental friendliness and low cost. Thus, Zn-based MOF materials and their composites have been extensively utilized in the detection of various pollutants, biomolecules and food additives. The Zn-MOF-based materials have been extensively utilized in electrochemical and fluorescence sensing applications. Previously, various Zn-MOF-based sensing systems such as pristine Zn-MOF, carbon-supported Zn-MOF composites, MXene hybrids with Zn-MOF, and bimetallic/trimetallic Zn-based MOFs were explored to enhance sensing performance. Such materials exhibit remarkable analytical performance, such as a low limit of detection (LOD) (nM to pM range), wide linear response range (LR), fast response times, and high selectivity in the presence of interfering species. In electrochemical sensing, Zn-MOF-modified electrodes demonstrated improved charge-transfer kinetics and sensitivity, enabling accurate determination of the biomolecules, drugs and heavy metal ions in real samples. Similarly, Zn-MOF-based fluorescence sensors showed high luminescent properties and displayed sensitive detection of pollutants and biomolecules. Despite such promising sensing performances, some challenges, such as low stability, reproducibility and selectivity in real-time monitoring, etc., remain that need to be overcome. This review article summarizes the previously reported literature on the fabrication of Zn-MOFs, their composites and Zn-MOF-derived materials for the development of electrochemical and fluorescence sensors. We have also discussed the future directions for the rational design of the high-performance Zn-MOF-based sensing systems for environmental and biomedical applications. We believe that the present review article would be useful for the scientific community working on the fabrication of Zn-MOF-based sensors.

## 1. Introduction

Industrialization, urban expansion and increasing global population have increased the release of hazardous pollutants and biologically relevant species into environmental and physiological systems [1,2]. The accurate detection of toxic metal ions, heavy metal ions, pharmaceutical residues, and disease biomarkers has therefore become a critical requirement in environmental monitoring, food safety, and clinical diagnostics [3,4,5]. The conventional methods, such as chromatography and spectroscopy, provide high precision, but their reliance on sophisticated instrumentation, labor-intensive procedures, and centralized laboratory settings significantly limits their applicability for on-site and real-time analysis [6,7,8]. Therefore, there is a need to develop advanced sensing platforms that are not only highly sensitive and selective but also portable, cost-effective, and operationally simple.

**Figure 1:**
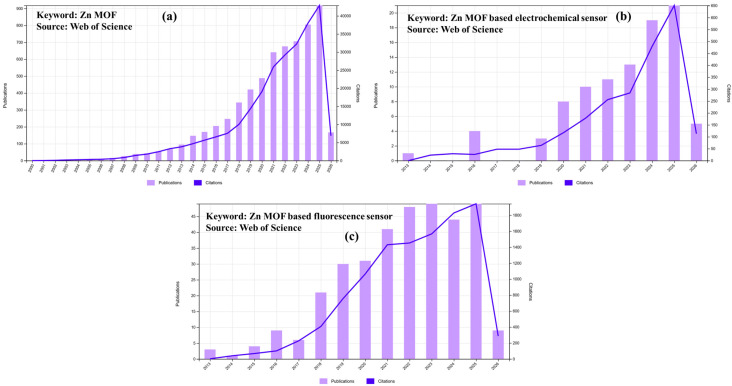
The bar graph shows the reported literature on Zn-MOF with different keywords (**a**–**c**). Source: Web of Science.

Recently, electrochemical and fluorescence-based sensing systems have emerged as promising candidates to address such challenges. Electrochemical sensors are particularly attractive due to their rapid response, high sensitivity, low detection limit and compatibility with miniaturized devices [9,10]. Similarly, fluorescence sensors also offer distinct benefits such as visual detectability, high signal-to-noise ratio, and suitability for non-invasive and real-time monitoring [11,12,13]. However, the performance of the electrochemical and fluorescence sensors depends on the properties of the active sensing materials [14,15,16]. Thus, the rational design and engineering of functional materials with tailored physicochemical properties remains a challenge for advancing next-generation sensor technologies [17,18,19]. Figure 1a–c show the previously reported literature on Zn-MOF-based materials for sensing applications.

Metal–organic frameworks (MOFs) have received significant attention as a promising class of porous crystalline materials composed of metal nodes interconnected by organic linkers [20,21]. It is worthy to mention that the 2025 Nobel Prize in Chemistry was awarded to Susumu Kitagawa, Richard Robson and Omar M. Yaghi for the development of MOFs. Previously, Robson et al. [22,23] have reported the synthesis of MOF materials. Similarly, Yaghi et al. [24] also reported hydrothermal synthesis of MOF containing large rectangular channels. Kitagawa et al. [25] reported the synthesis and crystal structure of a novel infinite-sheet and -chain copper (I) complex polymers. Owing to their exceptionally high surface area, tunable pore architectures, and structural diversity, MOFs provide a unique platform for the development of high-performance sensors [26]. In particular, zinc-based MOFs (Zn-MOFs) have emerged as promising candidates due to their low toxicity, environmental friendliness, facile synthesis and flexible coordination environments [27,28,29,30]. The past decade has witnessed significant progress in the application of Zn-MOFs for various electrochemical [31,32,33], luminescent [34], photo-catalytic [35], and hydrogen evolution applications [36]. Their intrinsic porosity and abundance of active sites facilitate efficient analyte adsorption and diffusion, whereas their structural tunability allows for precise functionalization to enhance selectivity. Moreover, the integration of Zn-MOFs with conductive materials such as carbon nanotubes, graphene derivatives, and emerging two-dimensional (2D) materials (e.g., MXenes) has led to the formation of hybrid architectures with synergistically improved electrical conductivity and catalytic activity [37,38]. Such advancements have substantially elevated the performance of electrochemical sensors, enabling ultra-low detection limits and wide linear response ranges for various target species.

Beyond electrochemical applications, Zn-MOFs have also demonstrated significant potential in fluorescence-based sensing [39]. Their luminescent behavior, arising from ligand-centered or metal-to-ligand charge-transfer processes, enables sensitive detection through fluorescence quenching or enhancement mechanisms [40]. These properties have been effectively exploited for the detection of nitroaromatic compounds, metal ions, antibiotics, and biomolecules with high selectivity and rapid response.

In this review, we have comprehensively summarized the recent advances in Zn-MOF-based materials for electrochemical and fluorescence sensing applications. Furthermore, we discuss the current limitations and future perspectives, providing insights to guide the rational development of next-generation Zn-MOF-based sensing platforms.

## 2. Synthesis Methods

Various synthesis methods such as hydrothermal, solvothermal, slow evaporation, mechanochemical, electrochemical, microwave, ultrasonic and sonochemical have been developed for the synthesis of MOF-based materials. In this section, we have briefly described the widely used synthesis method for the fabrication of MOF-based materials.

### 2.1. Hydrothermal/Solvothermal

**Scheme 1:**
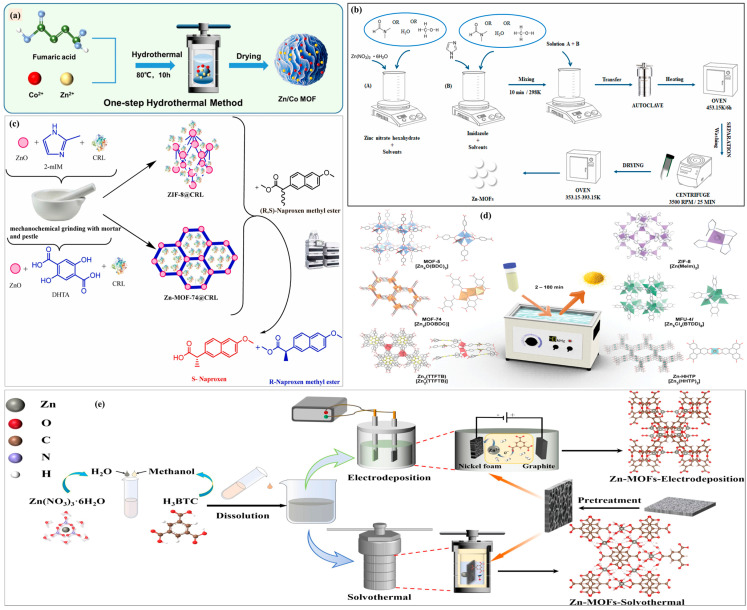
Synthetic approaches for MOF-based materials: (**a**) hydrothermal synthesis of ZnCo-MOF, (**b**) solvothermal synthesis of Zn-MOF, (**c**) mechanochemical synthesis of Zn-MOF-based materials, (**d**) sonochemical synthesis of MOF, and (**e**) electrodeposition synthesis of Zn-MOF. Reprinted with permission [42,43,47,50,57].

The hydrothermal and solvothermal methods are almost similar, except for the use of solvents. In the hydrothermal method, water has been widely used as a solvent for the synthesis of the MOF, whereas organic solvents are used in the solvothermal method. The hydrothermal/solvothermal method has various advantages such as control of reaction time, temperature, desired crystal size and uniform morphology. Fathima et al. [41] prepared Zn-MOF using the hydrothermal method by employing zinc nitrate hexahydrate as a Zn precursor. The prepared reaction precursor was transferred to the Teflon-lined autoclave, which was heated for 48 to 72 h at 100 °C. In another previous study, Zhang et al. [42] reported a one-step hydrothermal method for the fabrication of binary Zn-based MOF (ZnCo-MOF). The authors dissolved 8 mM fumaric acid, 1 mM zinc carbonate basic (3Zn(OH)_2_·2ZnCO_3_) and 2 mM cobalt chloride hexahydrate (CoCl_2_·6H_2_O) in 30 mL *N*,*N*-dimethylformamide (DMF) to obtain the reaction solution. This prepared reaction solution was transferred to the autoclave system and heated for 10 h at 80 °C (Scheme 1a). The obtained product was washed with methanol/ultrapure water to remove the residual particles or impurities. Alnafisah et al. [43] investigated the effects of different solvents for the preparation of Zn-MOF using the solvothermal method. The schematic graph in Scheme 1b shows the preparation of Zn-MOF. These reports indicate that hydrothermal and solvothermal methods are promising approaches for the preparation of Zn-based MOF materials.

### 2.2. Microwave

It is well-known that microwave-assisted synthesis methods involve the use of microwave radiation, which may significantly speed up the chemical reaction. In the microwave synthesis method, temperature, pressure, and volume of the reaction solutions can be varied to optimize the reaction conditions for better yield and improved properties. The MOF-based materials can be obtained within 0.5 h at 120 °C. Lu et al. [44] reported the fabrication of MOF-5 using a microwave-assisted synthesis method. In another study [45], a microwave-assisted ionothermal approach was also used for the preparation of ZIF-8. The microwave-assisted solvothermal method was also utilized for the preparation of ZIF-8 [46]. The reported literature shows that the microwave approach has potential for the synthesis of Zn-based MOF materials.

### 2.3. Mechanochemical

Previously, Ozyilmaz et al. [47] adopted the mechanochemical synthesis method for the preparation of Zn-based MOF materials. The schematic picture for the synthesis of Zn-based MOF has been displayed in Scheme 1c. The authors prepared lipase (CRL) molecules encapsulated in Zn-based MOFs (MOF-74 and ZIF-8) through a mechanochemical method. Zhao et al. [48] also used the mechanochemical method for the preparation of Zn-based MOF materials. The prepared materials displayed decent crystalline nature and phase purity. Nikmehr et al. [49] also adopted the mechanochemical ball milling method for the synthesis of Zn-based MOF materials. The above-mentioned reports indicate that the mechanochemical approach is cost-effective and an efficient approach for the synthesis of Zn-MOF materials.

### 2.4. Sonochemical

The sonochemical synthesis approach is well-known for its simplicity and sustainable sonochemistry for the preparation of MOFs. The sonochemical approach is more effective than the traditional approaches. The sonochemical method is based on the use of high-frequency ultrasonic irradiation to accelerate the chemical reactions in the solution. During the ultrasonication process, acoustic waves produce alternating compression and rarefaction cycles in the reaction medium, thereby resulting in the formation, growth, and implosive collapse of microbubbles, which is known as acoustic cavitation. The collapse of these bubbles generates localized hot spots with transient high temperatures and pressures, thereby enhancing nucleation, promoting metal–ligand coordination and accelerating crystal growth. Thus, the sonochemical synthesis method reduces reaction time and facilitates the formation of nanosized MOF particles compared to the conventional heating-based methods. The synthesis method has been illustrated in Scheme 1d [50]. Previously, the sonochemical method was widely used for the synthesis of MOF-based materials [50,51,52]. In particular, Vaitsis et al. [53] reported the fabrication of Zn-MOF using the sonochemical method. The synthesized materials were explored for the electrochemical reduction of carbon dioxide. Bigdeli et al. [54] also explored the potential of the sonochemical synthesis method for the fabrication of Zn-MOF. The above observations indicate the potential of the sonochemical synthesis method for the formation of Zn-MOF.

### 2.5. Electrochemical

The electrochemical method is one of the widely used synthesis methods for the formation of uniform morphological features or thin films on conductive substrates [55,56]. Previously, Zn-MOF was synthesized using the electrochemical (also called electrodeposition) method [57]. The schematic illustration for the synthesis of Zn-MOF via the electrochemical method has been displayed in Scheme 1e.

### 2.6. Slow Evaporation

The slow evaporation method is one of the widely used methods for the synthesis of MOFs [58]. The slow evaporation method also does not require any external energy supply [59]. Zaamouchi et al. [60] adopted the slow evaporation method for the formation of Zn-MOF for photo-catalytic applications. However, the slow evaporation approach needs more time to grow the crystal structures of MOF materials. It is worthy to mention that each approach has its unique advantages and limitations. Thus, it would be of great significance to summarize the advantages and limitations of the different synthesis methods. Table 1 shows the advantages and disadvantages of the various synthesis methods for the preparation of MOF materials.

## 3. Electrochemical Sensors

Recent years have witnessed the rapid growth in the fabrication of electrochemical sensors for the determination of various pollutants or biomolecules. MOF-based materials offer several advantages for the construction of electrochemical sensors. Previously, Zn-MOF-based materials were explored for the construction of electrochemical sensors for environmental monitoring and biomedical applications. Herein, we have summarized the previously reported electrochemical sensors based on Zn-MOF materials.

### 3.1. Zn-MOF and MOF/Metal Oxide-Based Sensors

**Figure 2:**
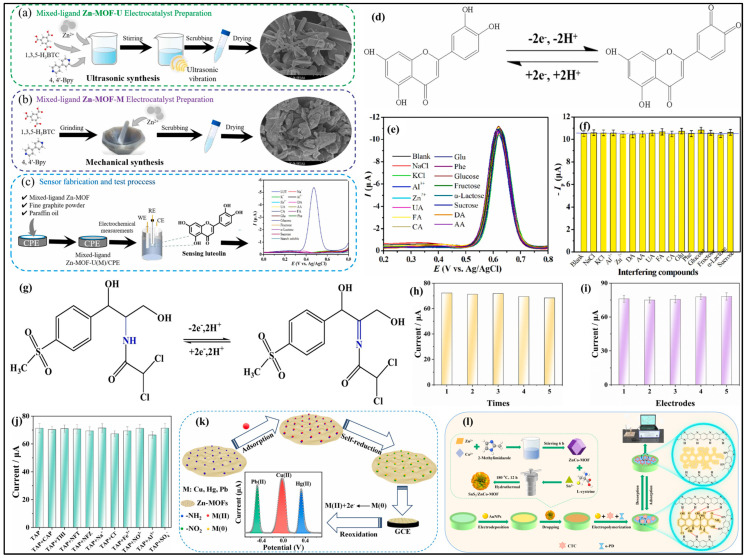
Schematic representation of the synthesis of Zn-MOF-U using the ultrasonication method (**a**) and Zn-MOF-M using the mechanical synthesis approach (**b**). (**c**) The schematic graph shows the fabrication of Zn-MOF-based CPE for LUT detection. (**d**) Electrochemical oxidation mechanism for LUT detection. (**e**) DPV curves and (**f**) histogram for selectivity results. (**g**) Reaction mechanism for the detection of TAP. DPV responses of the SXNU-1-Zn/GCE for (**h**) repeatability and (**i**) reproducibility. (**j**) Selectivity results for TAP detection. (**k**) Schematic illustration for the adsorption and detection of Cu (II), Hg (II) and Pb (II) using Zn-MOF. (**l**) Schematic graph shows the fabrication of the AZG sensor. Reproduced with permission [62,64,65,66].

Zn-MOF is one of the promising candidates for electrochemical applications due to its reasonable surface and physicochemical properties. Previously, Zn-MOF was adopted as a sensing material for the determination of chloroamphenicol (CAP) [61]. The authors polished the glassy carbon electrode (GCE) and sonicated it in the presence of ethanol and water for at least 10 min. Furthermore, an appropriate amount of Zn-MOF (5 µL (1 mg/mL)) was drop casted on GCE surface. Furthermore, Zn-MOF/GCE was immersed in deoxyacetic acid solution with o-phenylenediamine (O-PD) and CAP. The 15 cycles were run using cyclic voltammetry (CV), and the modified electrode was then placed into the methanol/ultrapure water solution and stirred for 8 min to remove the CAP template molecule. The modified electrode can be labeled as MIP/Zn-MOFs/GCE, which was explored as a CAP sensor. The developed sensor displayed interesting performance for CAP detection in terms of detection limit, sensitivity and real-sample applicability. In another study, Xie et al. [62] reported the fabrication of mixed-ligand-based Zn-MOF using simple synthetic protocols (ultrasonication = U and mechanical = M). The formation of the Zn-MOF using ultrasonication and mechanical methods has been illustrated in Figure 2a and Figure 2b, respectively. The obtained Zn-MOF-U and Zn-MOF-M displayed high specific surface areas of 55 m^2^/g and 187 m^2^/g, respectively. The synthesized mixed-ligand-based Zn-MOF (U or M) materials were adopted as the sensing material for the determination of luteolin (LUT). Before the detection of LUT, the active surface area of the Zn-MOF-U and Zn-MOF-M modified carbon paste electrode (CPE) was determined by using the Anson equation. The electrode surface area of the Zn-MOF-U@CPE and Zn-MOF-M@CPE was found to be 0.0287 cm^2^ and 0.02333 cm^2^, respectively. However, bare CPE displayed a surface area of 0.0121 cm^2^, which is lower compared to the Zn-MOF-U@CPE and Zn-MOF-M@CPE. The fabrication of the Zn-MOF-U (or M) modified CPE for LUT detection has been described in Figure 2c. The authors used differential pulse voltammetry (DPV) as the detection technique for the quantification of LUT. The obtained results indicated that Zn-MOF-U@CPE is a promising candidate for the monitoring of LUT and delivered a limit of detection (LOD) of 0.003 µM and a linear range (LR) of 0.005 to 10 µM using the DPV method. The oxidation of the LUT at the surface of the Zn-MOF-U-modified CPE has been illustrated in Figure 2d. The Zn-MOF-modified CPE also displayed excellent selectivity for LUT detection in presence of various interfering substances such as Al^3+^, Na^+^, Mg^2+^, K^+^, dopamine (DA), ascorbic acid (AA), uric acid (UA), folic acid (FA), citric acid (CA), phenylalanine (Phe), glutamic acid (Glu), glucose, α-lactose, fructose, and sucrose (Figure 2e,f). The proposed LUT sensor also displayed an excellent recovery rate of 97.9% to 102% in Honeysuckle tea and 99.5% to 103% in Duyiwei soft capsule samples. Therefore, it indicates that Zn-MOF-modified electrodes have the potential for practical applications. Ismail et al. [63] also explored the sonochemical synthesis method for the preparation of Zn-MOF towards the determination of paracetamol (PCM). The authors used a benzene dicarboxylate ligand for the formation of Zn-MOF. The authors also used a glassy carbon electrode (GCE) as the working substrate, and its active surface area was modified with the synthesized Zn-MOF as an electrode modifier. The Zn-MOF-modified GCE displayed an LOD of 0.104 μM with an LR of 1 to 50 μM for PCM detection. The improved performance of the Zn-MOF-modified GCE for PCM detection was ascribed to the presence of the porous framework and the high specific surface area of the Zn-MOF. Moreover, the strong adsorption capability of the Zn-MOF may accelerate the charge-transfer kinetics at the electrode–electrolyte interface, which may enhance the detection of PCM with reasonably good selectivity. Zhang et al. [64] reported the fabrication of thiamphenicol (TAP) sensor by employing two-dimensional (2D) Zn-MOF as sensing material. The Zn-MOF ({[Zn_3_(4, 4′-bpy)(1, 3, 5-BTC)_2_(DMPU)_2_(H_2_O)_4_]}_n_ (SXNU-1-Zn, Shanxi Normal University)) was prepared by using *N*,*N*′-dimethylpropenylurea (DMPU) as a templating agent. The layered 2D structure of Zn-MOF (SXNU-1-Zn) was formed by self-assembly of the triangular ligand 1, 3, 5-benzenetricarboxylic acid (1, 3, 5-BTC) and the linear ligand 4, 4′-bipyridine (4, 4′-bpy) with Zn ions. The SXNU-1-Zn was deposited on the GCE surface and employed as an electrochemical sensor for TAP detection. The authors obtained an LOD of 4.55 nM and an LR of 0.05 to 10 µM under the optimized conditions. The mechanism for TAP detection is illustrated in Figure 2g. The authors also observed that the proposed SXNU-1-Zn/GCE has decent repeatability (Figure 2h) and reproducibility (Figure 2i). The authors also reported excellent selectivity results for TAP detection in the presence of various interfering species (Figure 2j). These observations indicate that 2D Zn-MOF-based electrode modifiers are promising candidates for electrochemical sensing applications. Thus, another study also explored the potential of Zn-MOF as a sensing material for the construction of an electrochemical sensor for the determination of heavy metal ions such as copper, mercury, and lead (Cu (II), Hg (II) and Pb (II)) ions using square wave anodic stripping voltammetry (SWASV) [65]. The Zn-MOF was prepared by using 2, 4, 6-triaminopyrimidine as an organic ligand. The schematic graph for the determination of Cu (II), Hg (II) and Pb (II) is shown in Figure 2k. The obtained results displayed LOD of 0.17 µg/L, 0.25 µg/L and 0.22 µg/L for the detection of Cu (II), Hg (II) and Pb (II), respectively. Although this study reported reasonable LOD values for the determination of heavy metal ions, it lacks in-depth mechanistic aspects due to the involvement of adsorption studies. Future studies may consider in-depth investigations on the development of Zn-MOF-based sensors for heavy metal ion detection. It is well-known that residues of chlortetracycline (CTC) in food samples and products may present health risks to human beings. Therefore, the determination of CTC in food samples is of great significance for human safety. Thus, a novel composite of tin sulfide (SnS_2_) and ZnCo-MOF was prepared under benign conditions [66]. The prepared composite was combined with gold nanoparticles (Au NPs) to form the hybrid composite (Figure 2l). The molecularly imprinted polymer-based sensor was developed (Au-MIP/SnS_2_/ZnCo-MOF/Au/GCE = AZG) to determine the CTC in milk and egg samples. The proposed delivered LOD of 0.072 nM and LR of 0.1 to 100 µM for CTC detection. These results show that Zn-MOF-based materials offer promising properties and electrochemical performance for the development of sensors.

### 3.2. Zn-MOF/Carbon-Based Sensors

**Figure 3:**
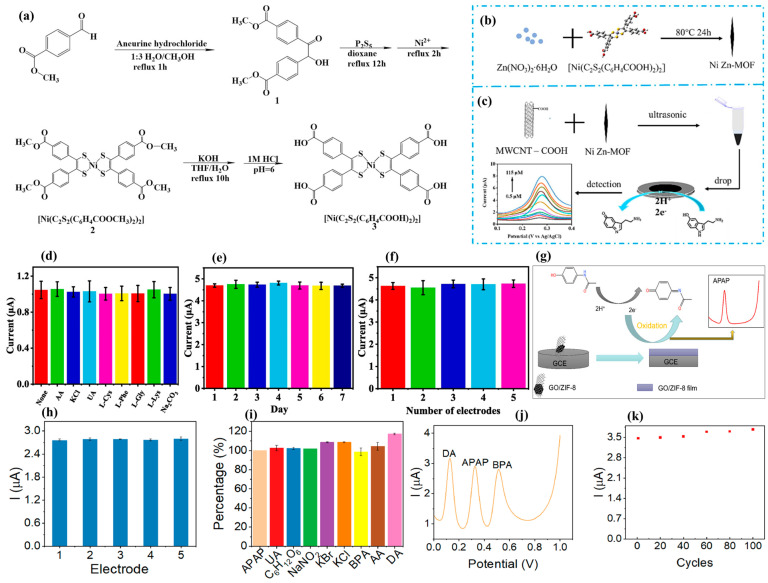
(**a**) Synthesis of the ligand. Preparation of (**b**) NiZn-MOF and (**c**) working of NiZn-MOF/MWCNTs/GCE for sensing of 5-HT. (**d**) Selectivity, (**e**) stability and (**f**) reproducibility results for 5-HT detection using NiZn-MOF/MWCNTs/GCE. Reproduced with permission [47]. (**g**) Reaction mechanism for AC detection using a GO/ZIF-8 sensor. (**h**) Reproducibility, (**i**) interference study, (**j**) selectivity, and (**k**) stability results for AC detection using the GO/ZIF-8 sensor. Reproduced with permission [68].

It is understood that carbon-based materials such as graphene or carbon nanotubes (CNTs), etc., have high surface area and interesting electrical properties. The incorporation of carbon-based materials with Zn-MOF may enhance the catalytic activities towards the determination of the targeted analyte. In this connection, a previous study demonstrated the incorporation of multi-walled carbon nanotubes (MWCNTs) into the Zn-based MOF material [67]. A novel ligand was prepared (Figure 3a) to form the Zn-MOF (Figure 3b). The authors fabricated Ni-Zn-MOF/MWCNTs/GCE for the determination of 5-Hydroxytryptamine (5-HT; serotonin) (Figure 3c). The authors also found that the prepared 5-HT sensor exhibits high selectivity (Figure 3d), stability (Figure 3e) and reproducibility (Figure 3f). The Ni-Zn-MOF/MWCNTs/GCE displayed enhanced electrical conductivity, which may facilitate the electron transfer kinetics of the modified electrode towards the detection of 5-HT. Therefore, authors obtained LOD of 0.03 μM and LR of 0.5 μM to 115 μM for 5-HT detection. Moreover, recovery studies exhibit reasonably good detection of 5-HT in urine samples. It is clear that the presence of carbon materials with Ni-Zn-MOF may have synergistic interactions and boost the electrochemical sensing performance of the Zn-MOF/MWCNTs/GCE for 5-HT detection. In another study [68], a graphene oxide (GO)/Zn-MOF (ZIF-8)-based composite was also fabricated for the detection of acetaminophen (AC). The DPV responses of the GO/ZIF-8/GCE were recorded in the presence of various concentrations of AC, and it was observed that the current response increases with increasing concentration of the AC. Moreover, GO/ZIF-8/GCE exhibited higher current response compared to ZIF-8/GCE. This shows that the presence of GO improved the electron capability of the ZIF-8 material. The reaction mechanism for AC detection is depicted in Figure 3g. The LOD of 0.014 μM and LR of 0.05 μM to 1.3 μM were obtained for AC detection using GO/ZIF-8/GCE. The reproducibility, interference study, selectivity, and stability results for AC detection are displayed in Figure 3h, Figure 3i, Figure 3j, and Figure 3k, respectively. This proposed electrode (GO/ZIF-8/GCE) was also found to be selective for AC detection in the presence of interfering substances. The real-sample applicability of the GO/ZIF-8/GCE was also examined in urine and pharmaceutical samples.

In another study [69], graphitic carbon nitride (g-C_3_N_4_) was also modified with Zn-MOF and deposited on a fluorine-doped tin oxide (FTO) substrate. The Zn-MOF/g-C_3_N_4_/FTO was explored as an electrode for the monitoring of naproxen (NPX). The Zn-MOF/g-C_3_N_4_/FTO displayed a low LOD of 2.3 ng/L and LR of 0.5 µg/L to 200 µg/L for NPX detection under the optimized conditions. Carbon-based materials are extensively used as a supporting material or conductive support to enhance the electron transfer and conductivity of various nanostructured materials. The amino functionalized MWCNTs (AMWCNTs) and methylene blue (MB)-modified Zn-MOF were also explored for the determination of human epidermal growth factor receptor 2 (HER2) biomarker [70]. The authors obtained an LOD of 0.05 ng/mL and excellent stability for 15 days. The aforementioned reports indicate that carbon-based materials are promising candidates for the preparation of Zn-MOF-based composites for electrochemical sensing applications.

### 3.3. Zn-MOF/MXene Based Sensors

**Figure 4:**
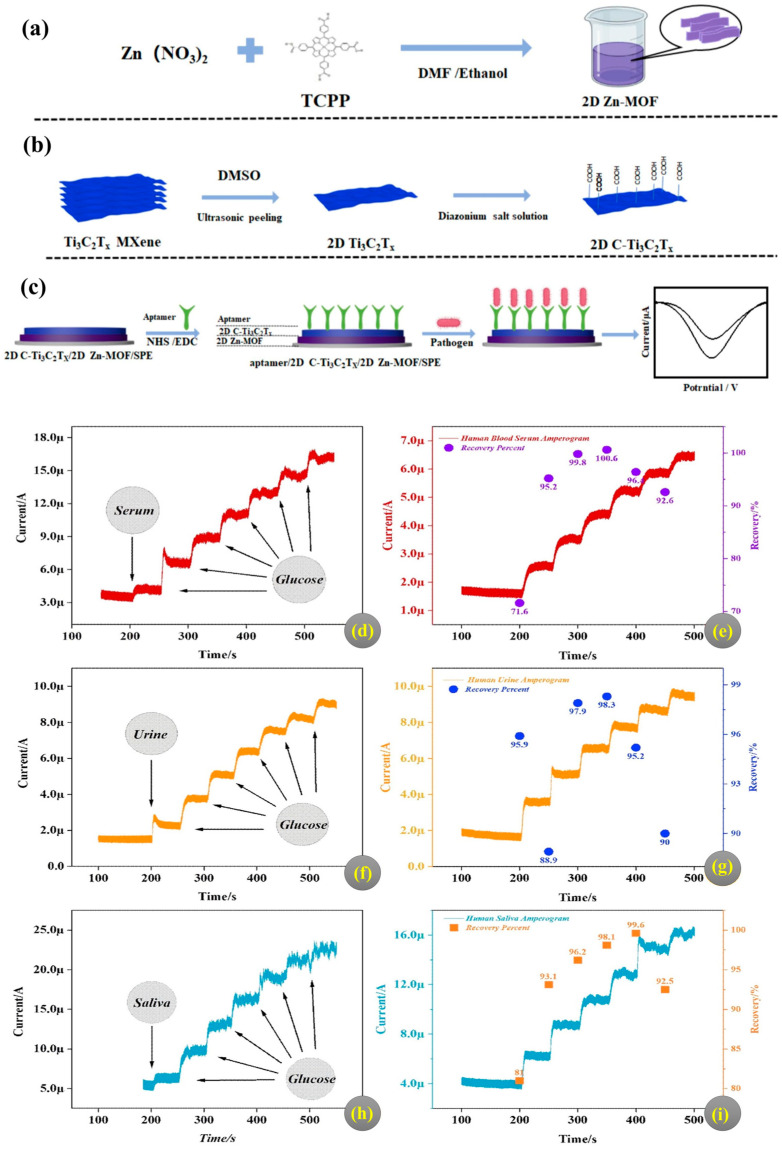
Schematic illustration of the preparation of (**a**) 2D Zn-MOF and (**b**) Ti_3_C_2_T_x_. (**c**) Pictorial representation of the sensing of a pathogen. Reproduced with permission [72]. Amperometric responses of the CoZn-MOF/GCE for the detection of glucose (glucose was added in the real samples) in real samples using the spike addition method: (**d**) human blood serum, (**f**) human urine and (**h**) human saliva samples. Amperometric response and recovery percentage for glucose addition and their recovery percent in (**e**) human serum, (**g**) human urine and (**i**) human saliva samples. Reproduced with permission [73].

Recently, MXenes have received tremendous interest from the scientific community due to their excellent conductivity and electronic properties. The introduction of MXene materials with Zn-MOF may be of great significance to improve the electrochemical detection of the targeted analyte. In this regard, titanium carbide (Ti_3_C_2_) MXene was integrated with Zn-MOF ([Zn_4_(btec)_2_(H_2_O)_6_]_n_·3nH_2_O and (H_4_BTC = 1, 2, 4, 5-benzenetetracarboxylate)) using a facile approach [71]. The Zn-MOF/Ti_3_C_2_/GCE was employed as a sensor for the detection of dopamine (DA). The Zn-MOF/Ti_3_C_2_/GCE displayed a reasonable LOD of 110 nM and an LR of 90 nM to 300 nM for DA detection. In addition, authors also found that Zn-MOF/Ti_3_C_2_/GCE is selective for the detection of DA in the presence of interfering species such as 5-aminovaleric acid (VA) and ascorbic acid (AA). This reveals that MXenes are promising supporting materials to enhance the conductivity and catalytic activities of the Zn-MOF-modified electrodes. In another study [72], two-dimensional (2D) carboxylated Ti_3_C_2_T_x_ (C-Ti_3_C_2_T_x_) MXene was also integrated with 2D Zn-MOF using benign synthetic protocols. The schematic graphs for the preparation of 2D Zn-MOF and 2D Ti_3_C_2_T_x_ are shown in Figure 4a and Figure 4b, respectively. The prepared composite material was explored for the monitoring of pathogens. The sensing process has been illustrated in Figure 4c. The aptamer/2D C-Ti_3_C_2_T_x_/2D Zn-MOF-modified screen-printed electrode (SPE) displayed reasonable catalytic behavior for the determination of *Escherichia coli* (*E. coli*), *Staphylococcus aureus* (*S. aureus*), and *Salmonella typhimurium* (*S. typhimurium*). The obtained results displayed LOD of 6, 5 and 5 CFU.mL^−1^ for the detection of *E. coli, S. aureus* and *S. typhimurium*, respectively. These results suggested that the incorporation of MXenes into the Zn-MOF materials enhanced the electrochemical activity of the modified electrodes. However, in-depth investigations of the mechanistic aspects need to be studied in the future.

### 3.4. Bimetallic and Trimetallic MOFs-Based Sensors

In the previous studies, bimetallic and trimetallic MOFs were also explored for the construction of electrochemical sensors. In this connection, a two-step synthesis approach was adopted for the fabrication of bimetallic CoZn-MOF for the development of non-enzymatic glucose sensors [73]. The CoZn-MOF was deposited on GCE, and its electrochemical response for glucose oxidation was checked using CV, amperometry (Amp) and chronoamperometry (CA) techniques. The authors found that the fabricated electrode has the potential to detect glucose with two wide LR of 0.001 mM to 0.255 mM and 0.255 mM to 2.53 mM and delivered a sensitivity value of 1218 μA.mM^−1^.cm^−2^ and 510 μA.mM^−1^.cm^−2^, respectively. In addition, the fabricated glucose sensor displayed an LOD of 4.7 μM, high selectivity in the presence of interfering species and decent recovery rate in human blood serum, saliva and urine samples. The real-sample recovery results for glucose detection have been displayed in Figure 4d–i. This indicated that bimetallic CoZn-MOF is one of the promising electrode materials for electrochemical sensing applications.

**Figure 5:**
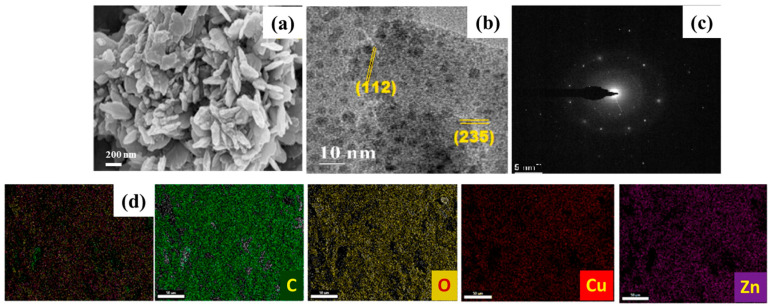
SEM (**a**) and HRTEM (**b**) images of Zn-Cu(TPA)MOF@GRP. (**c**) SAED image of Zn-Cu(TPA)MOF@GRP. (**d**) Elemental mapping results for the fabricated hybrid electrode. Reproduced with permission [76].

In another study [74], an electrochemical aptasensor was also developed for the determination of HER2. In this context, authors prepared ZnCo-MOF@ferrocene (Fc) and FeCo-MOF@MB materials for the construction of the HER2 sensor. The bimetallic ZnCo-MOF@Fc was prepared using an electrochemical method and modified with an aptamer, which acted as a capture probe. The FeCo-MOF was loaded with MB and utilized as a signal probe. The proposed electrochemical aptasensor displayed an LR of 0.75 pg/mL to 250 pg/mL and an LOD of 0.37 pg/mL. It can be noted that the proposed electrochemical aptasensor has the potential to detect breast cancer tumor biomarkers. The ZnCo-MOF was also explored for the determination of DA [75]. The ZnCo-MOF/GCE exhibits an LOD of 6.72 nM and an LR of 0.01 µM to 300 µM for DA detection using the DPV method. Moreover, authors investigated the selective nature of the ZnCo-MOF/GCE towards the monitoring of DA in the presence of uric acid (UA), AA, serine (Ser), glycine (Gly), and γ-aminobutyric acid (GABA). The observations revealed that ZnCo-MOF/GCE is highly selective for the determination of DA. The serum sample-based results also indicate the potential of ZnCo-MOF/GCE for practical applications. The ZnCu(terephthalic acid)-MOF was also integrated with graphite [ZnCu(TPA)MOF@GRP] for the construction of a cholesterol sensor [76]. The authors used SEM, high-resolution transmission electron microscopy (HRTEM), and energy-dispersive X-ray spectroscopy (EDX) mapping techniques to characterize the synthesized materials. The SEM image of the Zn-Cu(TPA)MOF@GRP is displayed in Figure 5a. The HRTEM images of the Zn-Cu(TPA)MOF@GRP are shown in Figure 5b. The selected area electron diffraction (SAED) of Zn-Cu(TPA)MOF@GRP is shown in Figure 5c. Furthermore, EDX mapping results were also obtained to confirm the formation of the Zn-Cu(TPA)MOF@GRP (Figure 5d). The obtained results authenticated the formation of Zn-Cu(TPA)MOF@GRP with decent phase purity.

**Figure 6:**
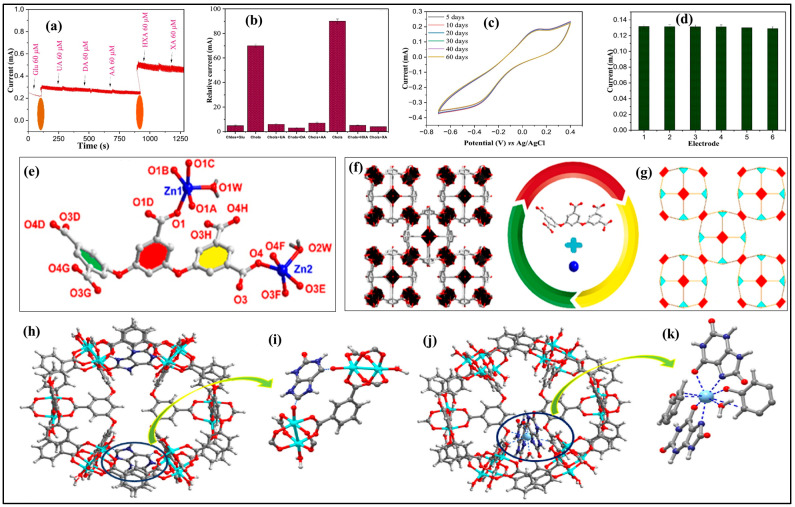
(**a**) Amperometric response of the ZnCu-MOF@GRP in the presence of interfering substances. (**b**) Selectivity results (bar graph). (**c**) CV results for stability test and (**d**) reproducibility results. Reproduced with permission [76]. (**e**) Coordination environment of the Zn-MOF. (**f**) 3D framework of Zn-MOF and (**g**) topology of Zn-MOF. Reproduced with permission [85]. (**h**,**i**) Interactions between Zn-MOF and UA and (**j**,**k**) Ce@Zn-MOF and UA. Reproduced with permission [85].

The ZnCu(TPA)MOF@GRP displayed a decent LOD of 0.028 μM, LR of 2.5 μM to 200 μM, and sensitivity of 333.33 μA.μM^−1^.cm^−2^, selectivity (Figure 6a,b), stability (Figure 6c) and reproducibility (Figure 6d). Moreover, this sensor exhibited a high recovery rate for the detection of cholesterol in milk samples. It is understood that heavy metal ions such as cadmium ions (Cd^2+^) are environmental pollutants that can cause significant harm to human health and ecological systems even at low concentrations. Therefore, a gold platinum nanoparticles (AuPt NPs) incorporated ZnNi-MOF-based sensor was developed for the monitoring of Cd^2+^ [77]. The authors observed that the proposed sensor displayed an LOD of 0.221 pM and an LR of 0.001 nM to 500 nM for the monitoring of Cd^2+^. In addition, excellent recovery of Cd^2+^ in tea, rice flour, liver powder, and tap water samples indicated the potential of the proposed sensor for practical applications. Abdel-Aziz et al. [78] also explored the potential of AlZn-MOF-based composite material for electrochemical sensing applications. The AlZn-MOF/MWCNTs/GCE was adopted as an electrochemical sensor for DA detection, and the obtained results displayed decent electro-catalytic behavior for the determination of DA. Ziaie et al. [79] also explored carbon paste electrode (CPE) modified reduced graphene oxide (rGO) and NiZn-MOF as an electrochemical sensor for diltiazem (DTZ) detection. The electrochemical impedance spectroscopy (EIS) studies revealed the presence of decent electrical conductivity in the fabricated electrode. The authors found that a fabricated electrochemical sensor has great potential to monitor the DTZ in human plasma, pharmaceutical tablets, and urine samples with acceptable recoveries. The presence of rGO provide larger surface area, enhanced the electrical conductivity and facilitates the electron transfer. Therefore, the fabricated sensor displayed enhanced electrochemical performance for DTZ detection. In another study, Yang et al. [80] also employed Au NPs@ZnCo-MOF/rGO as a sensing material for the determination of prostate-specific antigen (PSA). The content of Au NPs was varied to optimize the electrochemical performance of the proposed sensor for PSA detection. Thus, Au60NPs@ZnCo-MOF/rGO demonstrated LR of 100 fg/mL to 200 ng/mL and LOD of 60 fg/mL. Moreover, Au60NPs@ZnCo-MOF/rGO exhibited high selectivity, stability and reproducibility for PSA detection. Previously, an electrochemiluminescence (ECL) sensor was also developed for the determination of dinotefuran [81]. The authors prepared hollow PB-decorated biomass-derived carbon (BC) doped ZnCo MOF using a self-assembly approach. Furthermore, molecularly imprinted polymer (MIP) was prepared through the electro-polymerization approach by using *o*-aminophenol and dinotefuran as functional monomer and template molecule, respectively. The proposed sensor demonstrated LOD of 0.0046 µM and LR of 0.01 µM to 100 µM for the monitoring of dinotefuran. This sensor was also found to be highly selective, stable and reproducible for the monitoring of dinotefuran. In another study [82], a perfluorooctane sulfonic acid (PFOS) sensor was also fabricated using ZnTi-MOF integrated polymer-based material. The authors combined polypyrrole (PPy) with ZnTi-MOF for the construction of a PFOS sensor. The square wave voltammetry (SWV) technique was explored for the monitoring of PFOS. The PFOS sensor was constructed by modifying the CPE with magnetic MIP and ZnTi-MOF. The optimized conditions demonstrated an LOD of 0.7 nM and an LR of 0.002 µM to 165 µM for the sensing of PFOS. The real-time monitoring of PFOS was also evaluated by using spiked tap water, well water and river water samples, which exhibited acceptable recoveries. The lead ions (Pb^2+^) are the HMIs, which are highly toxic for human health and aquatic life. The ZnCu-BTC-NH_2_ MOF was adopted as a sensing material and deposited on the active area of the GCE [83]. The ZnCu-BTC-NH_2_ MOF/GCE exhibits improved electrochemical properties for the detection of Pb^2+^. The authors used square wave anodic stripping voltammetry (SWASV) for the detection of Pb^2+^ using ZnCu-BTC-NH_2_ MOF/GCE as a sensor. The LOD of 0.021 µg/L was obtained for Pb^2+^ detection. The CuZn-MOF was also incorporated with porous GO to form the hybrid composite [84]. The synthesized CuZn-MOF/PGO was modified with GPE and employed as a sensor for AA detection. The fabricated CuZn-MOF/PGO/PGE displayed a wide LR of 0.05 mM to 20 mM, LOD of 0.043 mM and excellent selectivity/stability for AA detection under the optimized conditions. Although this study demonstrated promising selectivity and stability for AA detection, poor LOD and real-sample studies in the presence of interfering substances should be evaluated in future studies. A novel Zn-MOF-based material with the introduction of Ce was also reported using the solvothermal method [85]. The coordination environment, 3D framework and topology of the Zn-MOF have been displayed in Figure 6e, Figure 6f and Figure 6g, respectively. The prepared Ce@Zn-MOF was deposited on the surface of GCE and utilized as a sensor for uric acid (UA) detection. The Ce@Zn-MOF/GCE exhibits interesting electrochemical performance for UA detection, which may be ascribed to the introduction of Ce ions that enhanced the charge-transfer capability of the Ce@Zn-MOF. The Ce@Zn-MOF-based electrode also shows high stability due to the presence of strong bonding interactions between the Ce ions and C/O atoms of the Zn-MOF. The interactions between Zn-MOF and UA have been displayed in Figure 6h,i, whereas those between Ce@Zn-MOF and UA are displayed in Figure 6j,k.

**Figure 7:**
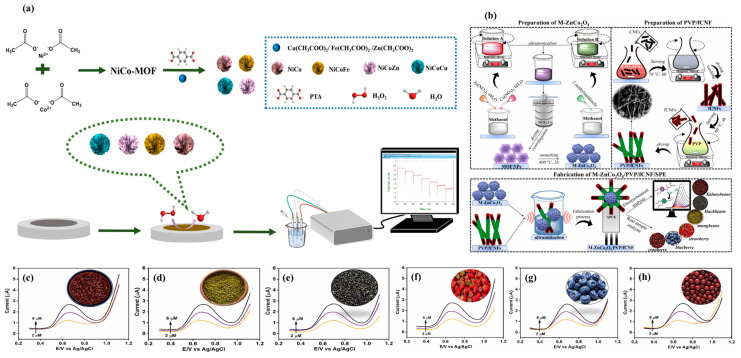
(**a**) Schematic illustration of the preparation of 2D NiCoM-MOFs (M = Zn, Fe or Cu) and application in an electrochemical sensor. Reproduced with permission [86]. (**b**) The schematic graph shows the preparation of M-ZnCo_2_O_4_, PVP/fCNF, and M-ZnCo_2_O_4_/PVP/fCNF/SPE. Reproduced with permission [90]. Real sample studies for DFC detection in (**c**) kidney beans, (**d**) mung beans, (**e**) black beans, (**f**) strawberries, (**g**) blueberries and (**h**) cranberries. Reproduced with permission [90].

Ma et al. [86] reported the facile preparation of trimetallic Zn-based MOF for the development of electrochemical sensors. A benign room temperature (RT) solution phase approach was utilized for the preparation of 2D NiCoM-MOFs (where M = Cu, Zn or Fe). The synthesis of the trimetallic MOFs and their application in a sensor has been illustrated in Figure 7a. The authors found that NiCoFe nanosheets have better electrochemical properties for the detection of hydrogen peroxide (H_2_O_2_). The authors observed that the presence of Fe in the NiCoFe-MOF plays a vital role in the detection of H_2_O_2_. The optimized conditions displayed an LOD of 2.1 μM and an LR of 5 μM to 15 mM for H_2_O_2_ detection. The real-sample studies in serum samples also displayed an acceptable recovery rate for H_2_O_2_ detection. It can be noted that NiCoFe-MOF was a highly efficient trimetallic MOF for H_2_O_2_ detection. We believe that further studies may consider the formation of a hybrid composite of Zn-based trimetallic MOF with conductive materials. In addition, NiCoZn-MOF can be used for the determination of other pollutants or biomolecules. The above-mentioned reports show that Zn-MOF-based bimetallic and trimetallic MOFs are promising candidates for electrochemical applications.

### 3.5. MOF-Derived Materials-Based Sensors

MOF-derived materials offer several advantages such as high surface area, porosity and catalytic activities. In this regard, Zn-MOF or bimetallic MOF-derived materials were extensively used in the development of electrochemical sensors. Manjula et al. [87] prepared a ZnCo-MOF-derived ZnCo catalyst for the detection of digoxin in urine samples. The ZnCo-MOF was prepared through the co-precipitation method followed by calcination. The obtained material was explored as an electrode modifier, and electrochemical studies such as DPV suggested that digoxin can be detected with an LOD of 0.0046 ng/mL. The practical applicability of the ZnCo-MOF-derived catalyst-based sensor was also validated using real urine samples, which showed satisfactory recovery rates. Shen et al. [88] reported the formation of MOF-derived zinc cobaltite (ZnCo_2_O_4_) integrated with chitosan-derived N-doped carbon (NC) composite. The ZnCo_2_O_4_@NC was applied as a sensing material for the monitoring of AC and p-aminophenol (p-AP). The ZnCo_2_O_4_@NC-based electrode displayed a wide LR of 8 µM to 520 µM and 6 µM to 420 µM for the detection of AC and p-AP, respectively. Interestingly, sensitivities of 0.1024 μA.μM^−1^.cm^−2^ and 0.2749 μA.μM^−1^.cm^−2^ were also obtained for the sensing of AC and p-AP, respectively. The real-sample studies were carried out using pharmaceutical tablets, which exhibited acceptable recovery rates in the range of 90% to 100%. The advantage of this study lies in the simultaneous detection of AC and p-AP. Divyarani et al. [89] also employed MOF-derived materials as sensing materials for the determination of glucose. The MOF-derived ZnCo_2_O_4_ was prepared and explored as an electrode modifier for the construction of a glucose sensor. The obtained results displayed an LOD of 24.8 nM and excellent long-term stability for glucose detection. Balram et al. [90] developed a novel electrochemical sensor for the detection of carbamate fungicide diethofencarb (DFC). The authors prepared a novel ternary composite of MOF-derived M-ZnCo_2_O_4_, functionalized carbon nanofibers (f-CNF), and amorphous non-ionic polymer polyvinylpyrrolidone (PVP). The preparation of M-ZnCo_2_O_4_, PVP/fCNF, and M-ZnCo_2_O_4_/PVP/fCNF/SPE has been illustrated in Figure 7b. The prepared M-ZnCo_2_O_4_/PVP/fCNF-based SPE was applied as a DFC sensor, which displayed an LOD of 2 nM and sensitivity of 5.21 μA.μM^−1^.cm^−2^. Moreover, authors achieved excellent recovery rates for the detection of DFC in strawberry, blueberry, cranberry, kidney beans, mung beans, and black beans samples. The voltammetric responses of the fabricated electrode (M-ZnCo_2_O_4_/PVP/fCNF/SPE) for DFC detection in kidney beans, mung beans, black beans, strawberries, blueberries, and cranberry samples have been depicted in Figure 7c, Figure 7d, Figure 7e, Figure 7f, Figure 7g, and Figure 7h, respectively. It is clear that the authors reported interesting findings and real-sample recovery for DFC detection in various samples. However, we believe that the sensitivity of this sensor should be further improved. Moreover, real-sample studies should be performed in the presence of various interfering substances.

### 3.6. Other Electrochemical Sensors

In some other studies, amine-functionalized MOFs (Fe-MOF, Ni-MOF, Zn-MOF, and Ti-MOF) were also synthesized through a solvothermal approach for the detection of chloramphenicol (CAP) [91]. Among the prepared materials, Fe-MOF exhibited the highest current response and enabled selective detection of CAP. The fabricated electrochemical sensor showed excellent sensitivity of 1.237 µA·µM^−1^·cm^−2^ with an LOD of 0.011 µM and LR of 0.04 µM to 68.18 µM. Furthermore, the sensor demonstrated a high detection efficiency of 98.9% for CAP analysis in milk and eye drop samples. Thus, it indicates that Zn-MOF is not a suitable candidate for the detection of CAP compared to the other MOF materials, or deeper mechanistic aspects should be studied in the future. The Cu BTC (BTC = benzene-1, 3, 5-tricarboxylate) and ZIF-8 (ZIF = zeolitic imidazolate framework) were also prepared and explored for the detection of nitrite ions using CV and CA techniques [92]. The LODs of 16.39 µM and 24.48 µM for nitrite ions were achieved using Cu BTC and ZIF-8, respectively. The LOD value for the ZIF-8-based sensor is poor compared to the Cu-BTC-based sensor. In the future, we may see some significant findings on the detection of ZIF-8-based nitrite sensors. The detection of rutin was also reported using a Zn-MOF-based MIP sensor, which displayed an LOD of 0.43 nM and an LR of 1 nM to 200 nM [93]. The above-mentioned reports indicate that Zn-MOF-based materials are interesting electrode materials for the construction of electrochemical sensors, but their performance should be further improved in terms of sensitivity, selectivity and long-term stability. The electrochemical performance of the sensors have been displayed in Table 2.

## 4. Fluorescence Sensors

**Figure 8:**
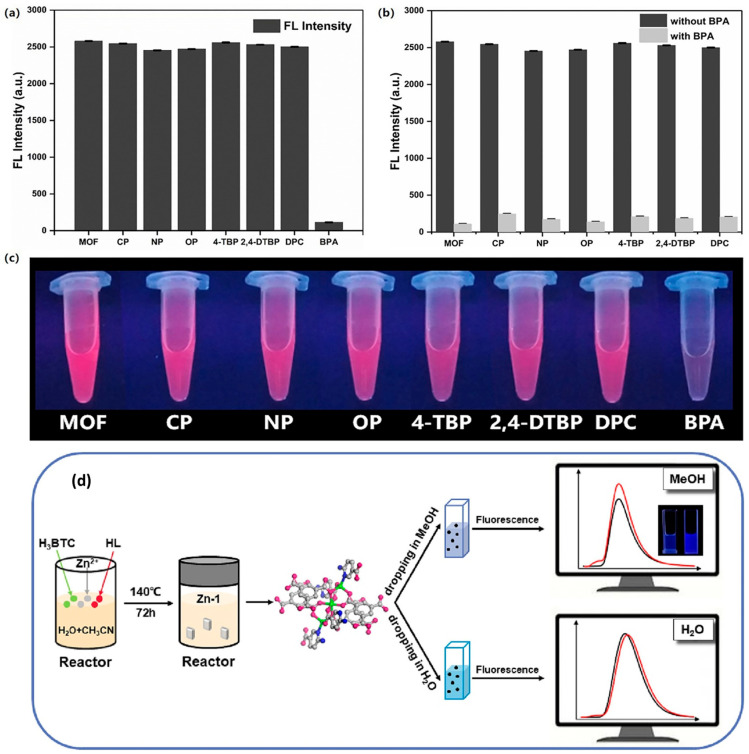
(**a**) Bar graph shows fluorescence intensity of Zn-TCPP-MOF before and after the addition of phenolic compounds. (**b**) Luminescence responses of the Zn-TCPP-MOF to BPA in the presence of other phenolic compounds. (**c**) Snapshots at 365 nm under UV light. Reproduced with permission [98]. (**d**) The schematic illustration shows the preparation of Zn-MOF and its application in a sensor. Reproduced with permission [100].

Although Zn-MOF-based materials have been extensively used in the construction of electrochemical sensors, various reports are available on the development of fluorescence sensors using Zn-MOF-based materials. The Zn-MOF materials are promising candidates for the construction of fluorescence sensors. In this connection, Shang et al. [94] synthesized [Zn_2_(deta)(bpy)]_n_ (1) (where H_4_deta = 4-(3, 5-dicarboxyphenoxy)phthalic acid and bpy = 2, 2′-bipyridine) using a benign synthetic approach. The Zn-MOF exhibited luminescent properties and can be used as a sensing material. Thus, prepared Zn-MOF was used as a sensing material for the determination of 2, 4, 6-trinitrophenol (TNP), aniline (ANI), tetracycline (TC), and lincomycin hydrochloride (LIN). It was also found that the determination of TNP and other nitroaromatic compounds can be distinguished by the naked eye due to the differences in fluorescence intensity under 365 nm ultraviolet (UV) irradiation. It can be stated that Zn-MOF materials are also promising materials for fluorescence sensing applications. In another study [95], a luminescent Zn-MOF ([Zn_2_(3-DBB)(1,4-bib)_1.5_(Ac)]·H_2_O (where 3-H_3_DBB = 3-(3, 5-dicarboxylato benzyloxy)benzoic acid and 1, 4-bib = 1, 4-bis(1-imidazoly)benzene)) was synthesized through hydrothermal method. The authors found that Zn ions were interconnected by 3-DBB^3−^ ligands to form the 2D brick-wall-type double-layer structure, which was extended into a 3D framework through pillar-like 1, 4-bib ligands. The synthesized Zn-MOF was also found to be selective for the determination of Dy^3+^ and delivered an LOD of 11.6 µM. The proposed Zn-MOF-based fluorescence sensor also exhibits LODs of 1.4 µM and 0.203 µM for nitrobenzene and aniline detection, respectively. The Zn-MOF was also employed as the sensing material for the determination of riboflavin (RF) using the fluorescence method [96]. The authors also examined the real-sample recovery of RF in milk and energy drink samples, which exhibited acceptable recovery rates in the range of 100.71% to 103.42%. Therefore, it suggests its potential for food industry applications. Kamal et al. [97] also developed a fluorescent sensor using Zn-based MOF materials for the determination of nitroaromatics and chromium (Cr^3+^) ions. In this connection, luminescent Zn-MOF ({[Zn_2_(pydc)_2_(DMF)]·1.5DMF}_n_) was synthesized using pyridine-2,5-dicarboxylic acid (H_2_pydc) as the organic ligand through the solvothermal method. The Zn-MOF-based fluorescent sensor displayed decent sensing capability for the determination of Cr^3+^. This sensor was also capable for the determination of TNP. In another previous work [98], Zn-MOF (Zn-TCPP) was employed as a bisphenol A (BPA) sensor. The bar graph shows fluorescence intensity of Zn-TCPP-MOF before and after the addition of phenolic compounds (Figure 8a), whereas corresponding snapshots under UV light at 365 nm are shown in Figure 8c. In addition, luminescence responses of the Zn-TCPP-MOF to BPA in the presence of other phenolic compounds have been displayed in Figure 8b. The authors observed that Zn-MOF has decent adsorption affinity towards BPA. The proposed BPA sensor displayed interesting sensitivity and high selectivity. Pang et al. [99] proposed a novel strategy for detecting harmful amines that migrate from packaging printing inks based on the fluorescence behavior of Zn-TCPE. The authors observed that the fluorescence of Zn-TCPE was quenched due to coordination with water molecules (in the aqueous solution). However, upon the addition of N-containing heterocyclic, aromatic, and aliphatic amines, competitive coordination substitution and electrostatic interactions with amino groups take place. Such interactions may restrict the motion of the aggregation-induced emission (AIE) active organic ligand, resulting in the restoration and enhancement of fluorescence. The proposed sensor also displayed decent detection limit and real-sample recovery, which suggested its potential for practical applications in amine detection. Hu et al. [100] prepared 2D Zn-MOF ({[Zn_3_(L^−^)_2_(HBTC^2−^)_2_]·2H_2_O·CH_3_CN}_n_), (H_3_BTC = 1, 3, 5-benzenetricarboxylic acid, HL = 6-aminonicotinic acid, and CH_3_CN = acetonitrile)) using the solvothermal method (Figure 8d). This Zn-MOF was explored as a D-tyrosine sensor, which displayed excellent selectivity and real sample recovery rates in milk samples.

**Figure 9:**
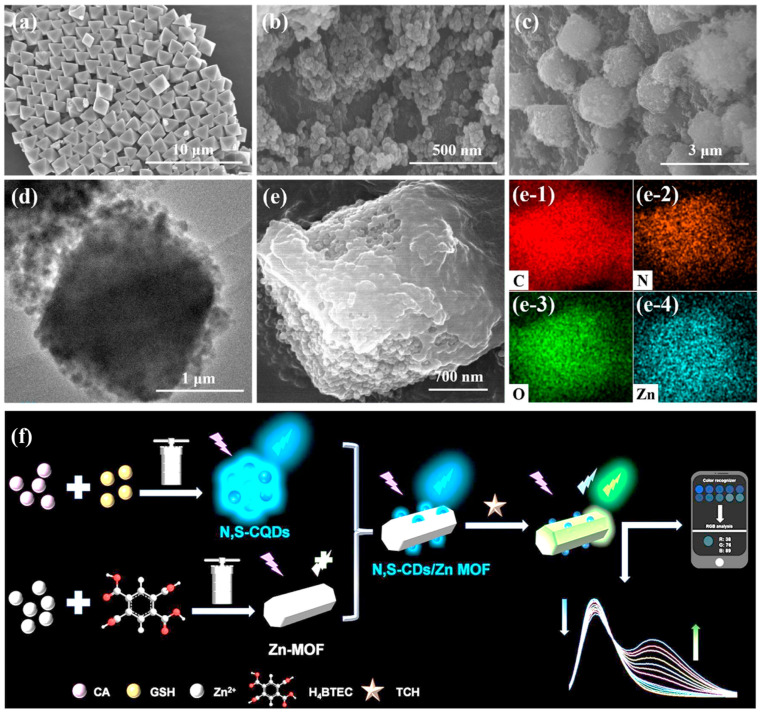
SEM pictures of (**a**) Znq_2_, (**b**) ZIF-8, (**c**) Znq_2_@ZIF-8. (**d**) TEM and (**e**) SEM-EDX mapping of Znq_2_@ZIF-8 ((**e-1**) = C, (**e-2**) = N, (**e-3**) = O and (**e-4**) = Zn). Reproduced with permission [106]. (**f**) Schematic picture for the fabrication of N, S-CDs/Zn-MOF and its application in a sensor. Reproduced with permission [117].

In another approach [101], the hydrothermal method was used for the preparation of Zn-MOF ([Zn_3_(μ_3_-OH)(L)·(H_2_O)]_n_, H_5_L = 3, 5-bis(3, 5-dicarboxyphenoxy)benzoic acid). Interestingly, the prepared Zn-MOF material exhibited remarkable selectivity and sensitivity towards the determination of the target analytes. The LODs of 4.10 μM and 6.63 μM were obtained for the detection of Cr_2_O_7_^2−^ and CrO_4_^2−^, respectively. The fluorescence sensing behavior of the Zn-MOF towards Cr_2_O_7_^2−^/CrO_4_^2^ ions may be attributed to the absorption between the analyte and the excitation/emission processes of the Zn-MOF. These obtained results suggested that Zn-MOF is an efficient and selective fluorescent sensor for the quantitative detection of Cr(VI) ions in aqueous environments. Zhao et al. [102] prepared tetra(4-carboxyphenyl) ethylene-based Zn-MOF (Zn-TCPE) for the detection of tobramycin (TOB). The Zn-TCPE-based fluorescence exhibits excellent selectivity for distinguishing aminoglycoside sulfate antibiotics. In another study [103], a novel Zn-MOF (Zn(Eu)-MOF@PAN NFM, PAN = polyacrylonitrile, NFM = nanofiber membrane) was also explored as a sensing material for the detection of nitrobenzene (NB), 4-nitrophenol (4-NP), benzaldehyde (BA), and Fe^3+^. The obtained results exhibited LOD of 0.861 ppm, 0.631 ppm, 0.981 ppm and 3.418 ppm for the detection of NB, 4-NP, BA, and Fe^3+^ respectively. Li et al. [104] reported the detection of gossypol using Zn-MOF. The Zn-MOF exhibited blue fluorescence, which enabled rapid and sensitive identification of gossypol through a fluorescence “turn-off” response. The proposed gossypol sensor demonstrated several advantages, such as a low LOD of 16.6 nM and high selectivity. Furthermore, its application in real samples such as cottonseed oil and fetal bovine serum exhibited decent performance with acceptable recovery of gossypol in the range of 99.35% to 101.5%. Therefore, it is suggested that a Zn-MOF-based fluorescent sensor may be used for food safety monitoring applications. In another study [105], a new multifunctional fluorescent sensor was developed using Zn-MOF as a sensing material. The Zn-MOF was prepared using zinc nitrate hexahydrate and pyridine-2, 5-dicarboxylic acid via the solvothermal method. The fluorescence sensing studies demonstrated that the prepared Zn-MOF exhibits high sensitivity toward Fe^3+^ and Al^3+^ ions. The LODs of 0.18 μM and 0.064 μM were obtained for the determination of Fe^3+^ and Al^3+^ ions, respectively. The authors proposed that the fluorescence quenching mechanism was attributed to the inner filter effect (IFE). In addition, the practical applicability of the proposed sensor was also authenticated in tap water samples. The obtained results displayed acceptable recoveries in the range of 103.0% to 107.9% for Fe^3+^ and 106.1% to 110.5% for Al^3+^ detection. Yang et al. [106] also explored the sensing properties of the Zn-based MOF materials for the detection of tetracycline (TC) in aqueous medium. The synthesized materials were characterized by SEM and EDX methods. The SEM and EDX results have been displayed in Figure 9a–e. Furthermore, prepared material was applied as a sensing material for TC detection. The obtained results displayed an LOD of 0.13 µM and high selectivity for TC detection. Madvar et al. [107] developed a raloxifene (RLX) sensor by utilizing fluorescent Zn-MOF material. The authors optimized the pH in the range of 3 to 10 to achieve the highest sensing performance of the proposed sensor. The proposed sensor was found to be stable and sensitive for RLX detection and delivered an LOD of 0.485 nM and an LR of 0.7 ng/mL to 350 ng/mL. Wang et al. [108] reported the synthesis of highly stable and luminescent Zn_5_-cluster-based MOF ({[Zn_5_(bci)_4_(OH)_2_(SO_4_)_2_]·4H_2_O}) using zwitterionic ligand H_2_bciCl (1, 3-bis(4-carboxybenzyl)-1H-imidazol-3-ium chloride) through the solvothermal method. The synthesized Zn-MOF was crystallized in the tetragonal crystal system with the P4_1_2_1_2 space group and features an 8-connected bcu body-centered cubic topology. It was also found that prepared MOFs exhibit high stability in water, various organic solvents, and a wide range of pH conditions. The synthesized Zn-MOF exhibited an LOD of 0.083 μM for TC detection. This sensor also displayed excellent recovery rates for TC detection in chicken, eggs, and fish samples, which suggested its potential for practical applications. The authors also proposed that the improved sensing mechanism was attributed to the synergistic contribution of fluorescence resonance energy transfer (FRET) and photo-induced electron transfer (PET). Zhang et al. [109] proposed the use of Zn-MOF as a sensing material for the determination of saccharin (SAC). The proposed SAC sensor displayed a fast response time of 30 s, LOD of 109 nM and LR of 0 to 160 μM. In another study [110], ZN-MOF ([Zn(TPPA)(L)]_n_) was prepared using π-electron-rich triangular ligand TPPA (TPPA = tris(4-(pyridin-4-yl)phenyl)amine) and H_2_L (H_2_L = 2, 3-dihydrothieno [3, 4-b][1,4]dioxine-5, 7-dicarboxylic acid). The photoluminescence investigations show that the emission of Zn-MOF originates from ligand-to-ligand charge-transfer transitions (π → π*). Owing to its strong luminescent properties, Zn-MOF functions as a multifunctional chemical sensor. The proposed sensing material shows interesting sensing behavior for the detection of nitroaromatic compounds, toxic metal ions, and antibiotics. Li et al. [111] reported two Zn-based MOFs ({[Me_2_NH_2_]_3_·[Zn_6_(L1)_2_·(H_2_O)_6_·μ_4_-O·μ_2_-O·HCOO]·3H_2_O·DMF}_n_ = (Zn-MOF1) and {[Me_2_NH_2_]_2_·[Zn_4_(L2)_2_·(H_2_O)_2_]·2H_2_O·DMF}_n_ = (Zn-MOF2)) through hydrothermal assisted synthesis method. The authors used pyridine–carboxylic acid ligands as L1 and L2 and synthesized Zn-MOF1 and Zn-MOF2, which demonstrated LODs of 2.821 mM and 3.581 mM for the detection of Fe^3+^ ions. Additionally, authors also found that Zn-MOF1 and Zn-MOF2 possess decent structural stability and repeatability after fluorescence sensing studies. In the previous report [112], a water-stable Zn-MOF ([Zn_3_(tzta)_2_(NMP)_2_(H_2_O)_2_]·2H_2_O) was also prepared using the solvothermal method. The carboxylate–tetrazolyl ligand H_2_tzta (2-(2-H-tetrazol-5-yl)-terephthalic acid) was utilized as a ligand for the formation of Zn-MOF. The authors observed that Zn-MOF and H_2_tzta ligand displayed similar emission peaks. In addition, Zn-MOF functioned as a turn-on fluorescent sensor for the monitoring of vitamin B_6_ (VB_6_) and cephalexin (CEP). It was also observed that Zn-MOF acted as a turn-off fluorescent sensor for Fe^3+^ detection. The LODs of 0.080 μM, 0.29 μM, and 0.021 μM were obtained for the detection of VB6, Fe^3+^, and CEP, respectively. The sensing mechanisms of the proposed sensor were attributed to FRET, internal filtration effect (IFE), and photon-induced electron transfer (PET). Hubale et al. [113] also proposed the synthesis of two novel Zn ([(Zn_2_(bib)(tdc)_2_(H_2_O)_2_)]_n_) and Cd-based MOFs ([Cd(bib)(tdc)(H_2_O)]_n_) using the solvothermal method. The prepared Zn-MOF and Cd-MOF exhibited decent selectivity and sensitive fluorescence sensing behavior towards the determination of Fe^3+^ and Cr_2_O_7_^2−^. The Zn-MOF and Cd-MOF-based sensors displayed LODs of 0.085 µM and 0.167 µM for the detection of Fe^3+^ ions. Similarly, Zn-MOF and Cd-MOF-based sensors displayed LODs of 0.037 µM and 0.045 µM for the detection of Cr_2_O_7_^2−^. In the previous study [114], a luminescent Zn-MOF ([Zn_3_(H_2_L1)_2_(OAc)_2_], H_2_L1 = (E)-N’-(3-ethoxy-2-hydroxybenzylidene) isonicotinohydrazide) was synthesized and employed as a sensing material for the detection of Fe^3+^ and Al^3+^. The authors found that the proposed sensor acted as a turn-off fluorescent sensor for the detection of Fe^3+^ and Al^3+^ and displayed excellent selectivity and sensitivity. The LODs of 0.1038 µM and 0.1857 µM were obtained for the detection of Al^3+^ and Fe^3+^, respectively. Liu et al. [115] reported the synthesis of 3D Zn(all-bdc)(Py) using the hydrothermal method. The synthesized Zn-MOF exhibited decent sensitivity towards Fe^3+^ and KMnO_4_^−^. This sensitivity may be ascribed to the presence of porous architecture and efficient ion interaction sites. The obtained results showed that LODs of 0.95 µM and 0.13 µM were obtained towards the detection of Fe^3+^ and KMnO_4_^−^, respectively. Feng et al. [116] developed a fluorescent sensor using a FRET system for the detection of RF. The Zn-MOF was integrated with graphitic carbon nitride quantum dots (g-C_3_N_4_ QDs) for RF detection. The synthesized g-C_3_N_4_ QDs/Zn-MOF exhibit decent sensitivity and selectivity for RF detection. The authors also found that synthesized g-C_3_N_4_ QDs/Zn-MOF has acceptable recovery of RF in milk and vitamin B_2_ tablets. The synthesized g-C_3_N_4_ QDs/Zn-MOF also displayed an LOD of 15 nM for RF detection. The improved performance may be ascribed to the presence of synergism in the synthesized g-C_3_N_4_ QDs/Zn-MOF composite. Sharma et al. [117] reported the fabrication of a ratiometric fluorescence sensor by using nitrogen- and sulfur-co-doped carbon dots (N, S-CDs)/Zn-MOF composite as sensing material (Figure 9f). The proposed sensor exhibits an LOD of 8.6 nM for TC detection. Furthermore, a smartphone-assisted agar slice platform was also developed to enable both visual and quantitative detection of TC. The authors proposed that the introduced device is cost-effective, portable, and user-friendly. In addition, this device displayed an LOD of 79 nM for TC detection with excellent sensitivity and practicality.

**Figure 10:**
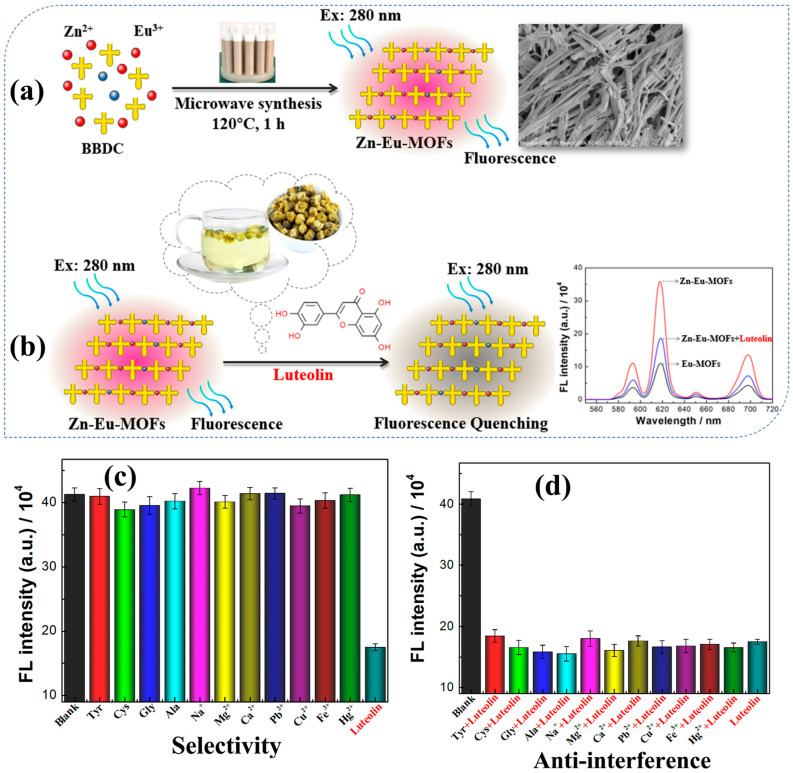
(**a**) The schematic graph shows the preparation of ZnEu-MOF and its application in the sensor (**b**). (**c**) Selectivity and (**d**) anti-interfering studies. Reproduced with permission [119].

Sharma et al. [118] also combined mesoporous carbon hollow spheres (MCHSs) with Zn-MOF for the determination of environmental pollutants such as Cu^2+^ and TNP. The synthesized Zn-MOF@MCHS displayed LODs of 0.368 µM and 0.301 µM for the detection of Cu^2+^ and TNP, respectively. In another research study [119], boric acid functionalized Zn-europium (Eu) binuclear MOF (Zn-Eu-MOFs) was also prepared (Figure 10a) and employed as a sensing material for the detection of luteolin in Chrysanthemum tea (Figure 10b). The proposed sensor exhibited an LOD of 16 nM for luteolin detection. The authors also obtained acceptable recovery of 99.5% to 102.7% and 99.3% to 100.6% in water and Chrysanthemum tea samples, respectively. The proposed sensor also displayed decent selectivity (Figure 10c) and anti-interfering properties (Figure 10d).

Wang et al. [120] developed a ciprofloxacin (CIP) fluorescent sensor using Zn-MOF-based materials. The Zn-MOF with TEMPO-oxidized cellulose nanofibers (TOCNF) (Zn-BDC@TOCNF) exhibited an LOD of 0.083 µM for CIP detection. The obtained results indicate that Zn-MOF-based materials are promising candidates for the detection of various pollutants and biomolecules. It is worthy to mention that sensing performance of Zn-MOF-based materials may be related to their structural and physicochemical properties. The high porosity and large surface area of Zn-MOF provide abundant adsorption sites and facilitate the diffusion of target analytes towards active centers. The Zn metal nodes and organic linkers can interact with analytes through coordination interactions, hydrogen bonding, π-π interactions, electrostatic attraction, or host-guest recognition, depending on the nature of the target molecule. In electrochemical sensing, these interactions improve analyte preconcentration at the electrode surface, whereas the incorporation of conductive materials such as rGO, MXenes, or conductive polymers enhances the electron-transfer kinetics and decreases charge-transfer resistance. Therefore, hybrid Zn-MOF composites generally show better sensitivity and LOD values compared to the pristine Zn-MOFs. In fluorescence sensing, Zn-MOF detects analytes through fluorescence quenching or enhancement caused by photo-induced electron transfer, energy transfer, ligand-to-metal charge transfer, and competitive absorption or framework–analyte interactions. It is believed that sensors with higher conductivity, decent water stability, better analyte-binding affinity, and more accessible active sites usually exhibit improved analytical performance in terms of low LOD values, wider linear ranges, higher selectivity, and better real-sample applicability. Thus, we believe that future work should study the depth of the mechanistic aspects. The sensing performance of the Zn-MOF-based fluorescence sensors has been summarized in Table 3.

## 5. Conclusions and Challenges

In summary, it is worthy to conclude that Zn-MOF-based materials have emerged as promising materials for electrochemical and fluorescence sensing applications. The presence of good porosity, larger specific surface area, flexible coordination environment and accessible active sites makes them suitable for the detection of biomolecules, pharmaceutical residues, toxic pollutants, and heavy metal ions. Moreover, the incorporation of Zn-MOF with conductive materials such as carbon nanostructures (graphene, graphitic carbon nitride and carbon nanotubes), MXenes, metal nanoparticles, metal oxides, and molecularly imprinted polymers has significantly improved their charge-transfer properties, catalytic activity, selectivity, and real-sample applicability. In fluorescence sensing, intrinsic or ligand-assisted luminescence of Zn-MOF has enabled sensitive detection via fluorescence quenching, enhancement, inner filter effect, photo-induced electron transfer and energy-transfer mechanisms. Despite these promising developments as mentioned above, several challenges still exist that may restrict the practical application of Zn-MOF-based sensors. The challenges and future perspectives have been mentioned below.

- Lack of an exact sensing mechanism.
- Most studies focus on analytical parameters only. For example, authors prepare the materials and modify the working electrode for the determination of the targeted analyte. The authors mainly focused on the calculations of sensing parameters such as LOD, linear range, and recovery values. Unfortunately, the authors did not focus on the long-term stability of more than 90 days and did not investigate the molecular-level interactions between the targeted analyte and the Zn-MOF framework.
- The electrical conductivity of the pristine Zn-MOF remained another challenge for the scientific community, although various reports demonstrated that incorporation of conductive supports such as carbon materials, polymers or MXenes, etc., may enhance the electrical conductivity of the Zn-MOF-based hybrid materials. This may also enhance the active sites, which improves the sensitivity of the Zn-MOF-based sensors.
- Stability issues under practical conditions.
- Reproducibility of sensor fabrication.
- The stability of the Zn-MOF-based sensors under real conditions needs to be studied in depth for practical applications.
- The sensing performance of the Zn-MOF-based sensors can be optimized through machine learning technology.
- The development of wearable and flexible sensors is of great importance for the future world. Therefore, future studies may consider such points.
- The scalability of the synthesis method should be improved.
- Long-term storage stability and reproducibility of the sensors under a real-time monitoring scenario should be carefully checked.
- Although many Zn-MOF-based sensors show acceptable selectivity under laboratory conditions, the selectivity of the Zn-MOF-based sensors should be studied in real samples.
- For practical application, future research should focus on the development of portable, miniaturized, and user-friendly sensing devices.

## Figures and Tables

**Table 1:** Advantages and limitations of the synthesis methods.

Synthesis Methods	Advantages	Limitations
Hydrothermal	Simplicity, single step reaction, less product damage, high purity, high yield and control over temperature, reaction time and surface morphology	High-pressure environment, needs particular equipment such as a Teflon-lined autoclave and high energy consumption
Solvothermal	Improved solubility of the organic linkers and metal ions, high yield, control over morphology, temperature and reaction time	High-pressure environment, organic solvents, need particular equipment such as a Teflon-lined autoclave and high energy consumption
Microwave	Fast and uniform heating, short reaction time, uniform particle size distribution	Need specific microwave equipment, scalability issues and high energy consumption
Mechanochemical	No need for solvents and environmentally friendly	Low yield and chances of unreacted particles
Sonochemical	Fast and environmentally friendly	Scalability
Electrochemical	Uniform film formation with controlled morphology	Toxic electrolytes
Slow evaporation	No external energy source	Time-consuming and low yield

**Table 2:** Electrochemical performance of the Zn-MOF sensors.

Material	LOD (µM)	Sensitivity (µA/(µM)·cm^2^)	Linear Range (µM)	Technique	Analyte	References
Mixed-ligand Zn-MOF-U/CPE	0.003	15.6 μA/mM/cm^2^	0.005–10.0	DPV	luteolin	[62]
GC/Zn-MOF	0.104	-	1–50	DPV	PCM	[63]
SXNU-1-Zn/GCE	0.00455	-	0.005–10	DPV	TAP	[64]
Au-MIP/SnS_2_/ZnCo-MOF/Au/GCE	0.072 nM	0.830 μA/μM	0.1–100	DPV	CTC	[66]
Ni Zn-MOF/MWCNTs	0.03	-	0.5–115	DPV	5-HT	[67]
GO/ZIF-8	0.014	-	0.05–1.3	DPV	APAP	[68]
MOF-Ti_3_C_2_/GCE	0.11	-	0.09–0.3	DPV	DA	[71]
2D C-Ti_3_C_2_T_x_ 2D Zn-MOF	6 CFU/mL	-	10^2^–10^7^ CFU/mL	DPV	*E. coli*	[72]
2D C-Ti_3_C_2_T_x_ 2D Zn-MOF	5 CFU/mL	-	10–1 × 10^8^ CFU/mL	DPV	*S. aureus*	[72]
2D C-Ti_3_C_2_T_x_ 2D Zn-MOF	5 CFU/mL	-	10–10^6^ CFU/mL	DPV	*S. typhimurium*	[72]
CoZn-BTC/GCE	4.7	1218 μA mM^−1^ cm^−2^, and 510 μA mM^−1^ cm^−2^	0.001–0.255 mM and 0.255–2.53 mM	Amperometry	Glucose	[73]
Zr-NDI/CNTs	0.6	-	0–100	DPV	DA	[75]
Zn-Cu(TPA)MOF@GRP	0.0028	-	2.5–200	DPV	Cholesterol	[76]
AuPt NPs@ZnNi-MOF	0.221 pM	-	0.001–500 nM	SWV	Cd^2+^	[77]
AlZn-BiMOF@MWCNTs/GCE	0.001	37.8	0.002–85	DPV	DA	[78]
Ni Zn MOF/rGO/CPE	0.002	-	0.009–0.090 and 0.100–10.00	DPV	DTZ	[79]
Au_60_NPs@Zn_1_Co_1_-MOF/rGO	0.06 pg/mL	-	0.0001–200 ng/mL	DPV	PSA	[80]
Zn/Cu-BTC-NH_2_/GCE	0.021 μg/L	-	0–120 μg/L	SWASV	Pb^2+^	[83]
Cu-Zn MOF/ PGO/GPE	0.043	-	0.05–20	CV	AA	[84]
NiCoFe	2.1	-	5–15000	i-t	H_2_O_2_	[86]
ZnCo-MOF	0.0046 ng/mL	-	0.02–0.38 nM	DPV	digoxin	[87]
ZnCo_2_O_4_@NC	0.2489	0.2749	6–420	DPV	PAP	[88]
ZnCo_2_O_4_@NC	0.0608	0.1024	8–520	DPV	APAP	[88]
ZnCo_2_O_4_@MOF	24.8 nM	-	0.1–100 mM	i-t	Glucose	[89]
M-ZnCo_2_O_4_/PVP/fCNF	2 nM	5.21	0.04–60.56	DPV	DFC	[90]
ZIF-8	16.39	2.91	0.05–1000	i-t	CA	[92]

**Table 3:** Sensing performance of the reported fluorescence sensors.

Material	LOD (µM)	Ksv (M^−1^)	Linear Range	Analyte	References
{[Zn_2_(H_4_deta)(2,2′-bpy)]_n_}	40.1 nM	-	-	ANI	[94]
{[Zn_2_(H_4_deta)(2,2′-bpy)]_n_}	74.1 nM	-	-	TC	[94]
{[Zn_2_(H_4_deta)(2,2′-bpy)]_n_}	141 nM	-	-	TNP	[94]
{[Zn(L)(H_2_O)]·DMF}_n_	20 nM	-	-	RF	[96]
{[Zn_2_(pydc)_2_(DMF)]·1.5DMF}_n_	0.12 ppb	2.1 × 10^8^	-	TNP	[97]
{[Zn_2_(pydc)_2_(DMF)]·1.5DMF}_n_	0.62 ppb	1.46 × 10^7^	-	Cr^3+^	[97]
Zn-TCPP-MOF	0.902	-	0.050–20 nM	BPA	[98]
Zn-MOF/MeOH (H_2_O) probe	79.70 × 10^−2^ μM	-	5–50 µM	Tyr	[100]
Zn(Eu)-MOF@PAN NFM	0.631 ppm	0.178 ppm^−1^	-	4-NP	[103]
Zn(Eu)-MOF@PAN NFM	0.981 ppm	0.051 ppm^−1^	-	BA	[103]
Zn(Eu)-MOF@PAN NFM	3.418 ppm	3.210 × 10^−3^ ppm^−1^	-	Fe^3+^	[103]
Zn(Eu)-MOF@PAN NFM	0.861 ppm	0.143 ppm^−1^	-	NB	[103]
Znq_2_@ZIF-8	0.13 μM	-	-	TC	[106]
{[Zn_5_(bci)_4_(OH)_2_(SO_4_)_2_]·4H_2_O}	0.083 μM	-	-	TC	[108]
{[Me_2_NH_2_]_3_·[Zn_6_(L1)_2_·(H_2_O)_6_·μ_4_-O·μ_2_-O·HCOO]·3H_2_O·DMF}_n_	2.821 × 10^−3^ M	2.858 × 10^3^ M^−1^	-	Fe^3+^	[111]
{[Me_2_NH_2_]_2_·[Zn_4_(L2)_2_·(H_2_O)_2_]·2H_2_O·DMF}_n_	3.581 × 10^−3^ M	9.245 × 10^3^ M^−1^	-	Fe^3+^	[111]
[Zn_3_(H_2_L^1^)_2_(OAc)_2_] (Zn-MOF)	0.1857 µM	-	-	Fe^3+^	[114]
[Zn_3_(H_2_L^1^)_2_(OAc)_2_] (Zn-MOF)	0.1038 µM	-	-	Al^3+^	[114]
Zn-MOF@MCHS	0.301 µM	0.36 × 10^6^ M^−1^	-	TNP	[118]
Zn-MOF@MCHS	0.368 µM	0.83 × 10^4^ M^−1^	-	Cu^2+^	[118]
Zn-BDC@TOCNF	0.083	-	-	CIP	[120]

## Data Availability

No new data was generated to describe this study.

## References

[1] Antenozio M.L., Caissutti C., Caporusso F.M., Marzi D., Brunetti P. (2024). Urban Air Pollution and Plant Tolerance: Omics Responses to Ozone, Nitrogen Oxides, and Particulate Matter. Plants.

[2] Varatharajan G.R., Ndayishimiye J.C., Nyirabuhoro P. (2025). Emerging Contaminants: A Rising Threat to Urban Water and a Barrier to Achieving SDG-Aligned Planetary Protection. Water.

[3] Xie X., Hu X., Cao X., Zhou Q., Yang W., Yu R., Liu S., Hu H., Qi J., Zhang Z. (2025). Heavy Metal Ion Detection Based on Lateral Flow Assay Technology: Principles and Applications. Biosensors.

[4] Mititelu M., Neacșu S.M., Busnatu Ș.S., Scafa-Udriște A., Andronic O., Lăcraru A.-E., Ioniță-Mîndrican C.-B., Lupuliasa D., Negrei C., Olteanu G. (2025). Assessing Heavy Metal Contamination in Food: Implications for Human Health and Environmental Safety. Toxics.

[5] Varga I., Bilandžić N., Morović S., Košutić K. (2026). Pharmaceuticals in Food and Water: Monitoring, Analytical Methods of Detection and Quantification, and Removal Strategies. Separations.

[6] Kidanemariam A., Cho S. (2025). Metal–Organic-Framework-Based Optical Biosensors: Recent Advances in Pathogen Detection and Environmental Monitoring. Sensors.

[7] Bahlol H.S., Li J., Deng J., Foda M.F., Han H. (2024). Recent Progress in Nanomaterial-Based Surface-Enhanced Raman Spectroscopy for Food Safety Detection. Nanomaterials.

[8] Albu C., Chira A., Radu G.-L., Eremia S.A.V. (2025). Advances in Cost-Effective Chemosensors for Sustainable Monitoring in Food Safety and Processing. Chemosensors.

[9] Sridev J., Deen A.R., Ali M.Y., Ting W.-T., Deen M.J., Howlader M.M.R. (2025). Advanced Electrochemical Sensors for Rapid and Sensitive Monitoring of Tryptophan and Tryptamine in Clinical Diagnostics. Biosensors.

[10] Mello G.A.B., Benjamin S.R., de Lima F., Dutra R.F. (2025). Recent Advances in Electrochemical Sensors for the Detection of Anti-Inflammatory and Antibiotic Drugs: A Comprehensive Review. Biosensors.

[11] Shi Y., Zhang W., Xue Y., Zhang J. (2023). Fluorescent Sensors for Detecting and Imaging Metal Ions in Biological Systems: Recent Advances and Future Perspectives. Chemosensors.

[12] Buonasera K., Galletta M., Calvo M.R., Pezzotti Escobar G., Leonardi A.A., Irrera A. (2025). Organic Fluorescent Sensors for Environmental Analysis: A Critical Review and Insights into Inorganic Alternatives. Nanomaterials.

[13] Hasan J., Bok S. (2024). Plasmonic Fluorescence Sensors in Diagnosis of Infectious Diseases. Biosensors.

[14] Bounegru A.V., Dinu Iacob A., Iticescu C., Georgescu P.L. (2025). Electrochemical Sensors and Biosensors for the Detection of Pharmaceutical Contaminants in Natural Waters—A Comprehensive Review. Chemosensors.

[15] Ahmad K., Suganthi S., Rajkumar C., Vignesh S., Gautam R.K.S., Oh T.H. (2026). Progress in Electrochemical and Fluorescence Sensors for Propyl Gallate Monitoring in Food Samples. Biosensors.

[16] Kakkar S., Gupta P., Kumar N., Kant K. (2023). Progress in Fluorescence Biosensing and Food Safety towards Point-of-Detection (PoD) System. Biosensors.

[17] Zheng B., Zhou H., Zhao G., Wang K., Wu P., Liu H., Wang P., Yao Y., Xu F. (2025). Bioinspired electrically conductive hydrogels: Rational engineering for next-generation flexible mechanosensors. Mater. Sci. Eng. R Rep..

[18] Machín A., Márquez F. (2025). Next-Generation Chemical Sensors: The Convergence of Nanomaterials, Advanced Characterization, and Real-World Applications. Chemosensors.

[19] Chang X., Fang Y., Ivasenko O. (2025). Towards a Rational Design of Biosensors: Engineering Covalently Grafted Interfacial Adlayers as a Testbed Platform for Electrochemical Detection of Epinephrine. Molecules.

[20] He L., Wang Z., Wang H., Wu Y.-N. (2025). Are MOFs ready for environmental applications: Assessing stability against natural stressors?. Coord. Chem. Rev..

[21] Li D., Yadav A., Zhou H., Roy K., Thanasekaran P., Lee C. (2024). Advances and Applications of Metal-Organic Frameworks (MOFs) in Emerging Technologies: A Comprehensive Review. Glob. Chall..

[22] Bell M., Edwards A.J., Hoskins B.F., Kachab E.H., Robson R. (1989). Synthesis and x-ray crystal structures of tetranickel and tetrazinc complexes of a macrocyclic tetranucleating ligand. J. Am. Chem. Soc..

[23] Hoskins B.F., Robson R. (1989). Infinite polymeric frameworks consisting of three dimensionally linked rod-like segments. J. Am. Chem. Soc..

[24] Yaghi O.M., Li H. (1995). Hydrothermal Synthesis of a Metal-Organic Framework Containing Large Rectangular Channels. J. Am. Chem. Soc..

[25] Kitagawa S., Munakata M., Tanimura T. (1992). Synthesis of the novel infinite-sheet and -chain copper(I) complex polymers {[Cu(C_4_H_4_N_2_)_3/2_(CH_3_CN)](PF)_6_)·0.5C_3_H_6_O}_∞_ and {[Cu_2_(C_8_H_12_N_2_)_3_](ClO_4_)_2_}_∞_ and their x-ray crystal structures. Inorg. Chem..

[26] Kidanemariam A., Cho S. (2025). Advanced Metal–Organic Framework-Based Sensor Systems for Gas and Environmental Monitoring: From Material Design to Embedded Applications. Sensors.

[27] Sharma S., Chand P., Kaushik S. (2024). A critical review of recent advancements in zinc based metal organic framework nanocomposites and their derivatives for supercapacitor applications with future perspectives and challenges. Sustain. Mater. Technol..

[28] Huang Q., Xie T., Luo Y., Zhou J.-E., Wu Y., Lin X., Yang H. (2025). A Comprehensive Review on Zinc-Based MOFs and Their Derivatives for Alkali-Ion Batteries: Synthesis, Applications, and Future Prospects. Adv. Funct. Mater..

[29] Capsoni D., Guerra G., Puscalau C., Maraschi F., Bruni G., Monteforte F., Profumo A., Sturini M. (2021). Zinc Based Metal-Organic Frameworks as Ofloxacin Adsorbents in Polluted Waters: ZIF-8 vs. Zn_3_(BTC)_2_. Int. J. Environ. Res. Public Health.

[30] Safdar Ali R., Meng H., Li Z. (2022). Zinc-Based Metal-Organic Frameworks in Drug Delivery, Cell Imaging, and Sensing. Molecules.

[31] Sun J., Zhang Q., Liu C., Zhang A., Hou L., Yuan C. (2024). Conductive Zinc-Based Metal–Organic Framework Nanorods as Cathodes for High-Performance Zn-Ion Capacitors. Batteries.

[32] Rani S., Sharma B., Kapoor S., Malhotra R., Varma R.S., Dilbaghi N. (2019). Construction of Silver Quantum Dot Immobilized Zn-MOF-8 Composite for Electrochemical Sensing of 2,4-Dinitrotoluene. Appl. Sci..

[33] Humayanthvarma C.A., Kumar G., Meenatchi G.K. (2026). Synthesis and electrochemical characterization of zinc (Zn) based metal organic framework (MOF) and its polymer composite with pyrrole and RuO_2_ for supercapacitor application. J. Mater. Sci. Mater. Electron..

[34] Rani P., Husain A., Bhasin K.K., Kumar G. (2024). Zinc(II)-MOF: A Versatile Luminescent Sensor for Selective Molecular Recognition of Flame Retardants and Antibiotics. Inorg. Chem..

[35] Yuan C., Miao Y., Chai Y., Zhang X., Dong X., Zhao Y. (2023). Highly Water-Stable Zinc Based Metal–Organic Framework: Antibacterial, Photocatalytic Degradation and Photoelectric Responses. Molecules.

[36] Dang L.-L., Zhang T.-T., Li T.-T., Chen T., Zhao Y., Zhao C.-C., Ma L.-F. (2022). Stable Zinc-Based Metal-Organic Framework Photocatalyst for Effective Visible-Light-Driven Hydrogen Production. Molecules.

[37] Huang H., Qin J., Liu C., Luo L., Lan Y., Yang L., Zhang J., He H. (2024). Constructing Zn-based MOF-MXene nanoarchitectures to stabilize ultrafine Pt nanocrystals with enhanced methanol oxidation performance. Carbon.

[38] Bulin C., Guo T., Bao J. (2026). Zn-MOF-graphene oxide conjugate for neodymium(III) recovery: Performance optimization and mechanistic insights. Sep. Purif. Technol..

[39] Zavahir S., Ben Yahia H., Schneider J., Han D., Krupa I., Altamash T., Atilhan M., Amhamed A., Kasak P. (2022). Fluorescent Zn(II)-Based Metal-Organic Framework: Interaction with Organic Solvents and CO_2_ and Methane Capture. Molecules.

[40] Wang N., Li S., Li Z., Gong Y., Li X. (2023). A Zn(II)–Metal–Organic Framework Based on 4-(4-Carboxy phenoxy) Phthalate Acid as Luminescent Sensor for Detection of Acetone and Tetracycline. Molecules.

[41] Fathima A.F., Surya S., Bazeera A.Z., Roshan M.M., Mani R.J. (2025). Hydrothermal synthesis and structural characterization of zinc glutarate nanocrystals: A metal-organic framework study. Next Mater..

[42] Zhang M., Li J., Wu L., Liang T., Liu J., Wang L. (2026). One-step hydrothermal synthesis of Zn/Co MOF for efficiently activating PMS to degrade organic pollutants in water: The reaction kinetics and mechanism. Sep. Purif. Technol..

[43] Alnafisah M.S., Alharbi K.N., Almuqati N.S., Almalahi K.M., Almusawa M.H., Almotairy D.D., Alotaibi G., Alromaeh A.I. (2025). Effect of solvent selection on Zn-MOFs synthesized for hydrogen storage applications. Int. J. Hydrogen Energy.

[44] Lu C.-M., Liu J., Xiao K., Harris A.T. (2010). Microwave enhanced synthesis of MOF-5 and its CO_2_ capture ability at moderate temperatures across multiple capture and release cycles. Chem. Eng. J..

[45] Yang H., Lu L. (2012). Microwave-assisted Ionothermal synthesis and Characteriza-tion of Zeolitic Imidazolate Framework-8. Chin. J. Chem..

[46] Lai L.S., Yeong Y.F., Lau K.K., Shariff A.M. (2016). Effect of synthesis parameters on the formation of ZIF-8 under microwave-assisted Solvothermal. Procedia Eng..

[47] Ozyilmaz E., Caglar O. (2023). Rapid mechanochemical production of biocomposites by encapsulating enzymes to zinc based-two metal organic frameworks (ZIF-8 and Zn-MOF-74) for enantioselective hydrolysis reaction of racemic Naproxen methyl ester. Process Biochem..

[48] Zhao J., Liu Y., Xu T., Li W., Meng R.B., Tang L.L., Liu D.D., Xu F., Deng N.M. (2026). Mechanochemical synthesis of metal-organic frameworks: An efficient green pathway and mechanistic investigation. J. Solid State Chem..

[49] Nikmehr S., Kazemzad M., Sabzehmeidani M.M., Nikzad L., Ebadzadeh T., Mousaei M. (2023). Zn-based MOFs materials fabricated by mechanochemical ball milling and hydrothermal method and derived metal oxides. Results Chem..

[50] Yi J., Lee G., Park S.S. (2024). Solvent-Induced Structural Rearrangement in Ultrasound-Assisted Synthesis of Metal–Organic Frameworks. Small Methods.

[51] Eliwa A.S., Ali A.E., Hosny W.M., Mohamed G.G., Deghadi R.G. (2023). Sonochemical synthesis and characterization of novel copper based metal-organic framework: Its application as electrochemical sensor for determination of Cd(II) ion in real water samples. Inorg. Chem. Commun..

[52] Lee Y.-R., Cho S.-M., Ahn W.-S., Lee C.-H., Lee K.-H., Cho W.-S. (2015). Facile synthesis of an IRMOF-3 membrane on porous Al2O3 substrate via a sonochemical route. Microporous Mesoporous Mater..

[53] Vaitsis C., Kanellou E., Pandis P.K., Papamichael I., Sourkouni G., Zorpas A.A., Argirusis C. (2022). Sonochemical synthesis of zinc adipate Metal-Organic Framework (MOF) for the electrochemical reduction of CO_2_: MOF and circular economy potential. Sustain. Chem. Pharm..

[54] Bigdeli M., Morsali A. (2015). Sonochemical synthesis of a nano-structured zinc(II) amidic pillar metal–organic framework. Ultrason. Sonochem..

[55] Ren H., Wei T. (2022). Electrochemical Synthesis Methods of Metal-Organic Frameworks and Their Environmental Analysis Applications: A Review. ChemElectroChem.

[56] Zhou S., Shekhah O., Jia J., Czaban-Jóźwiak J., Bhatt P.M., Ramírez A., Gascon J., Eddaoudi M. (2021). Electrochemical synthesis of continuous metal–organic framework membranes for separation of hydrocarbons. Nat. Energy.

[57] Cao Y.-J., Chen Z.-Y., Peng H.-L., Chen H.-P., Xie C.-F., Fan J.-P. (2025). One-step electrochemical synthesis of Zn-MOFs/NF composite electrodes for efficient electrosorption of organic dyes. Sep. Purif. Technol..

[58] Raptopoulou C.P. (2021). Metal-Organic Frameworks: Synthetic Methods and Potential Applications. Materials.

[59] Bedia J., Muelas-Ramos V., Peñas-Garzón M., Gómez-Avilés A., Rodríguez J.J., Belver C. (2019). A Review on the Synthesis and Characterization of Metal Organic Frameworks for Photocatalytic Water Purification. Catalysts.

[60] Zaamouchi I., Kaci M.M., Zidane Y., Belaid S., Bouacida S., Benmerad B. (2024). The impressive photocatalytic performance of Zn-MOF as a novel photocatalyst for the effective purification of dyes under solar exposure. J. Mol. Struct..

[61] Zhao Y., Wang R., Wang Y., Jie G., Zhou H. (2023). Dual-channel molecularly imprinted sensor based on dual-potential electrochemiluminescence of Zn-MOFs for double detection of trace chloramphenicol. Food Chem..

[62] Xie C., Li H., Niu B., Guo H., Lin X. (2024). Comparison of ultrasonic vs mechanochemistry methods for fabrication of mixed-ligand Zn-based MOFs for electrochemical determination of luteolin. J. Alloys Compd..

[63] Ismail K.M., Hassan S.S., Medany S.S., Hefnawy M.A. (2024). A facile sonochemical synthesis of the Zn-based metal–organic framework for electrochemical sensing of paracetamol. Mater. Adv..

[64] Zhang C., Yan B., Yang H., Liu L., Li S., Shi J., Wang B., Lei J., Fu Y., Ji W. (2025). Interface engineering of 2D Zn-MOF layers structures for thiamphenicol-specific electrochemical sensing. Microchem. J..

[65] Shao Z., Di K., Ding L., You F., Fan C., Wang K. (2025). Amino-enriched Zn-MOFs with self-reduction for energy-free simultaneous removal and electrochemical detection of heavy metal ions in the aquatic environment. Anal. Chim. Acta.

[66] Sun R., Han S., Zong W., Chu H., Zhang X., Jiang H. (2024). Ultrasensitive detection of chlortetracycline in animal-origin food using molecularly imprinted electrochemical sensor based on SnS_2_/ZnCo-MOF and AuNPs. Food Chem..

[67] Chen J., Yang J., Chen Y., Dong J., Deng R., Zhu L. (2023). Multiwalled carbon nanotubes modified with nickel-zinc bis(dithiolene) metal-organic frameworks for electrochemical detection of 5-hydroxytryptamine. J. Electroanal. Chem..

[68] Wang S., Chen F., Li Z., Tao H., Qu L., Li J., Zhu M., Zha Q. (2023). A graphene oxide/Zn-metal organic framework electrochemical sensor for acetaminophen detection. Surf. Interfaces.

[69] Ambaye A.D., Zikalala S.A., Mashiloane K.C., Nure J.F., Kebede M.A., Mokrani T., Nxumalo E.N. (2024). Development of engineered Zn-MOF/g-C3N4 based photoelectrochemical system for real-time sensors and removal of naproxen in wastewater. Talanta Open.

[70] Zhao P., Xu X., Li J., Zhou X., Zhao Z., Xuan C., Tian Q., Jumuddin F.A., Pan D. (2025). Ultrasensitive electrochemical biosensor for HER2 detection based on MB@ZnMOF prepared by a facile ultrasound-assisted bottom-up strategy. Microchem. J..

[71] Paul J., Kim J. (2023). Reticular synthesis of a conductive composite derived from metal-organic framework and Mxene for the electrochemical detection of dopamine. Appl. Surf. Sci..

[72] Yang L., Ding Y., Ma Y., Wen J., Wang J., Dai G., Mo F. (2025). An electrochemical sensor based on 2D Zn-MOFs and 2D C-Ti_3_C_2_T_x_ composite materials for rapid and direct detection of various foodborne pathogens. Food Chem..

[73] Kachouei M.A., Shahrokhian S., Ezzati M. (2021). Bimetallic CoZn-MOFs easily derived from CoZn-LDHs, as a suitable platform in fabrication of a non-enzymatic electrochemical sensor for detecting glucose in human fluids. Sens. Actuators B Chem..

[74] Zhang Y., Xu Y., Li N., Ma W., Yang M., Hou C., Huo D. (2023). An ultra-sensitive dual-signal ratio electrochemical aptasensor based on functionalized bimetallic MOF nanocomplexes by the in-situ electrochemical synthesis for detect HER2. Int. J. Hydrogen Energy.

[75] Tran V.A., Vo G.N.L., Doan V.D., Thanh N.C., Lam T.D., Le V.T. (2024). Modification sub-nano Zn-Co Metal-Organic framework for electrochemical detection of neurotransmitter. Microchem. J..

[76] Kushwaha K.S., Dey B., Ahmad M.W., Syed A., Wong L.S., Singh R., Mukherjee S.K., Yang D.-J. (2025). Arup Choudhury, Novel electrochemical enzyme-free sensor based on bimetallic zinc-copper MOF@graphite rod for the detection of cholesterol in milk sample. Microchem. J..

[77] Zhai L., Feng X., Jin H., Bai T., Wei M., Liu Y., Suo Z. (2025). An electrochemical sensor based on hydrangea like AuPt NPs@ZnNi-MOF combined with hyperbranched HCR signal amplification strategy for Cd^2+^ detection. Food Biosci..

[78] Abdel-Aziz A.M., Sidqi M.E., Radwan A., Sayed M.A., Aziz A.A.A. (2025). Highly sensitive voltammetric sensor for dopamine based on a novel bimetallic AlZn MOF@ multi-walled carbon nanotubes: Fabrication, electrochemical characterization and applications. Microchem. J..

[79] Ziaie N., Ghoreishi S.M. (2025). Novel electrochemical detection of diltiazem in the presence of amlodipine and acetaminophen using a NiZn MOF/rGO modified carbon paste electrode. Microchem. J..

[80] Yang Y., Cai Q., Wang W., Dai C., Hu K., Ren M., Xu H., Hu J. (2025). Construction and electrochemical performances of AuNPs functionalized bimetallic ZnCo-MOF derivatives and reduced graphene hybrid structure for highly sensitive prostate specific antigen detection. Microchem. J..

[81] Dai S., Chen H., Zhang Y., Zhang L., Liu T., Wu C., Sun M., Su G., Ye J., Wang Y. (2025). Enhanced sensing of dinotefuran in foods based on BC/ZnCo MOF@PBA nano-enzyme induced MIECL sensor. Food Chem..

[82] Rezaei M., Ghanavati M., Mohammadi N., Khani S., Nasirimoghadam S., Smiley E., Basiryanmahabadi A. (2024). A new sensitive layer based on clcinated Zn/Ti-MOF/magnetic molecularly imprinted polypyrrole: Application to preconcentration and electrochemical determination of perfluorooctane sulfonic acid by magnetic carbon paste electrode. Talanta.

[83] Qi T., Yuan Z., Meng F. (2024). Highly sensitive and highly selective lead ion electrochemical sensor based on zn/cu-btc-nh2 bimetallic MOFs with nano-reticulated reinforcing microstructure. Anal. Chim. Acta.

[84] Muhaddas A., Ijaz A., Abbas G., Hayat A., Rahim A., Hassan A. (2025). In-situ synthesis of bimetallic-MOF/porous graphene oxide nanocomposite for electrochemical sensing of ascorbic acid. Microchem. J..

[85] Zhang J., Gao L., Zhang Y., Guo R., Hu T. (2021). A heterometallic sensor based on Ce@Zn-MOF for electrochemical recognition of uric acid. Microporous Mesoporous Mater..

[86] Ma Y., Wei P., Chen M., Shi X., Lu X., Zhang X., Sun D. (2023). Trimetallic metal–organic framework nanosheets as nanozymes for the electrochemical sensing of H_2_O_2_. J. Electroanal. Chem..

[87] Manjula N., Pulikkutty S., Chen S.-M. (2023). Simple synthesis of MOF-derived Zn, Co electrocatalyst for sensitive detection of digoxin in urine sample. Colloids Surf. A Physicochem. Eng. Asp..

[88] Shen T., Zhou Y., Wu D., He H., Xie A., Luo S. (2024). MOF derived ZnCo_2_O_4_@nitrogen-doped carbon as an electrochemical sensor for simultaneous detection of acetaminophen and p-aminophenol. Diam. Relat. Mater..

[89] Divyarani K., Sreenivasa S., Kumar S., Vinod A., Alharethy F., Jeon B.-H., Devi V.S.A., Martis P., Parashuram L. (2024). Fabrication of a novel MOF template-derived ZnCo_2_O_4_ composite for the non-enzymatic electrochemical detection of glucose. Results Chem..

[90] Balram D., Lian K.-Y., Sebastian N., Alharthi S.S., Al-Saidi H.M. (2025). Synergy of MOF derived hydrangea-like ZnCo_2_O_4_ embedded PVP/fCNF tertiary nanocomposite for diethofencarb quantification in beans and berries. J. Alloys Compd..

[91] Dhayanithi C.A., Palpandi K., Raman N., Babu S.G. (2023). Development of amine-based transition metal MOFs as efficient electrochemical sensors for the detection of chloramphenicol in food and pharmaceutical samples. Electrochim. Acta.

[92] Nagasaka T., Gopalram K., Nagashree K.L., Venkatesan S. (2025). Electrochemical sensing of nitrite by Cu and Zn based metal-organic frameworks—A green synthesis approach. J. Mol. Struct..

[93] Wang H., He Y., Wang R., Lu M., Luo Y., Sun Y., Xu D. (2025). An electrochemical sensor of PBA-grafted Zn-MOF/MIP for sensitive rutin detection. Microchem. J..

[94] Shang L., Chen X.-L., Liu L., Hou X., Cui H.-L., Yang H., Wang J.-J. (2021). Novel multifunctional Zn Metal−Organic framework fluorescent probe demonstrating unique sensitivity and selectivity for detection of TNP, ANI, TC and LIN in water solution. J. Solid State Chem..

[95] Yang X., Ren Y., Hou X., Wang Z. (2021). A fluorescent 1,4-bib-pillared Zn-MOF sensor for highly sensitive detection of Dy^3+^, nitrobenzene and aniline in aqueous solution. J. Solid State Chem..

[96] Fan L., Li J., Sun C., Zhang J., Zhao Y., Li W., Chang Z. (2022). An ultra-sensitive fluorescent sensor based on Zn-MOF for selective detection of riboflavin in food. J. Solid State Chem..

[97] Kamal S., Khalid M., Khan M.S., Shahid M., Ahmad M. (2022). A Zinc(II) MOF for recognition of nitroaromatic explosive and Cr(III) ion. J. Solid State Chem..

[98] Pang Y., Cao Y., Han J., Xia Y., He Z., Sun L., Liang J. (2022). A novel fluorescence sensor based on Zn porphyrin MOFs for the detection of bisphenol A with highly selectivity and sensitivity. Food Control.

[99] Pang Y., Li S., Wang H., Zhang N., Chen R., Tan C.S., Xia Y., Zhao H., Cao Y., Liang J. (2023). Small-molecular amines fluorescence sensor based on the destruction of an aggregation-induced-emission-active Zn metal-organic framework. J. Solid State Chem..

[100] Hu Y.-Y., Wu X.-Q., Shi H.-P., Wei X.-H. (2023). 2D Zn-MOF fluorescence probe for detecting D-tyrosine in methanol or aqueous systems. Microchem. J..

[101] Yang S., Chen S., Li K., Luo Q., Zhang Y., Wang L., Wu S., Zhu M. (2023). A water-stable metal–organic framework Zn-MOF as a chemical sensor for efficient detection of Cr(VI) (Cr_2_O_7_^2−^ and CrO_4_^2−^) anions in water. Inorganica Chim. Acta.

[102] Zhao Y., Hao H., Wang H., Sun L., Zhang N., Zhang X., Liang J. (2023). Antibiotic quantitative fluorescence chemical sensor based on Zn-MOF aggregation-induced emission characteristics. Microchem. J..

[103] Chen M., Shao R., Wang Q., Gao Y., Ma Y., Guan R., Yang T. (2023). Eu doped Zn-MOF nanofiber fluorescent membrane and its multifunctional detection of nitroaromatic compounds and Fe^3+^. Polyhedron.

[104] Li W., Zhu J., Qi X., Sun C., Li W., Chang Z. (2024). A novel Zn-MOF fluorescent probe for highly sensitive and rapid detection of gossypol in cottonseed oil and fetal bovine serum. Food Biosci..

[105] Mao X., Li H., Shi Y., Liu J., Kuai L., Yang F., Wu C. (2024). A multifunctional fluorescence sensor based Zn(II) metal-organic framework for rapid and sensitive detection Fe^3+^ and Al^3+^. Polyhedron.

[106] Yang L., Xu T., Zou H., Zhang S., Huang J., Cai L., Zou D., Huang J., Yao M. (2024). Self-assembled fluorescent Zn-MOF with high specific surface area based on the coordination interaction for sensitive detection and selective removal of tetracycline antibiotic in water. Opt. Mater..

[107] Madvar R.R., Taher M.A. (2024). Preparation of fluorescent sensor based on Zn metal-organic framework for detection and determination of raloxifene as an anticancer drug. Environ. Res..

[108] Wang K., Dong Y., Zhao X., Bai X., Li L., Guo J., Wang Z., Tang H., Ma Y. (2024). Highly sensitive fluorescence detection of tetracycline in food samples using a Zn5 cluster-based zwitterionic metal-organic framework. J. Mol. Struct..

[109] Zhang E., Zhang G., Lu W., Liu D., Li A., Zhang Q., Jiang L., Ju P., Qu F. (2025). Equipment-free detection of saccharin based on the confined space enhanced AIE effect of a novel ratiometric fluorescent Zn-MOF: Probe design, sensing performance, and practical applications. Sens. Actuators B Chem..

[110] Ren H.-R., Liu Y., Zhang Y.-T., Yan P.-J., Qin D.-D., Yao X.-Q. (2025). A novel Zn MOFs as a multifunctional sensitive chemical sensor for the rapid detection of nitroaromatic compounds, Tetracycline, Fe^3+^, and degradation for methyl orange. Inorganica Chim. Acta.

[111] Li W.-L., Yan W.-W., Zheng L.-N., Xue N., Gong X.-S., Ding T. (2025). Two stable Zn(II) metal-organic frameworks used as fluorescence sensors for efficient Fe(III) detection. J. Solid State Chem..

[112] Wang X.-Q., Wang X., Zhu H., Xue Y., Yang J., Cui P., Jiao J. (2025). A water-stable Zn(II)-based metal-organic framework as a multifunctional fluorescent sensor for vitamin B6, Fe3+ ion and cephalexin. Spectrochim. Acta Part A Mol. Biomol. Spectrosc..

[113] Hubale V., Dalvi A., Nille O., Sadale S., Kolekar G., Sawant V. (2025). Zn(II)/Cd(II) MOFs based on 2,5-thiophenedicarboxylic acid and bis(Imidazole) linkers for highly selective and sensitive detection of Fe^3+^ and Cr_2_O_7_^2−^. J. Mol. Struct..

[114] De A., Singh V., Dhar J., Mishra S. (2025). Zn (II) based fluorescent metal-organic framework as a ‘turn-off’ sensor for Al^3+^ and Fe^3+^ ions in aqueous solution: Synthesis, DFT calculations and Hirshfeld surface analysis. J. Mol. Struct..

[115] Liu H., Zhao Y., Huang B., Liu H., Zhang P., Gu W., Ma T. (2025). Zn-Based Three-Dimensional Metal-Organic Framework for Selective Fluorescence Detection in Zwitterionic Ions. Int. J. Mol. Sci..

[116] Feng S., Pei F., Wu Y., Lv J., Hao Q., Yang T., Tong Z., Lei W. (2021). A ratiometric fluorescent sensor based on g-CNQDs@Zn-MOF for the sensitive detection of riboflavin via FRET. Spectrochim. Acta Part A Mol. Biomol. Spectrosc..

[117] Sharma I., Kumar A., Arya K., Mehra S., Kumar A., Mehta S.K., Kataria R., Han B.-M., Gao W.-J., Wang F.-Q. (2025). A ratiometric fluorescent sensor for sensitive and visual detection of tetracycline hydrochloride based on Zn MOF and N, S-doped carbon dots. Microchem. J..

[118] Sharma I., Kumar A., Arya K., Mehra S., Kumar A., Mehta S.K., Kataria R. (2025). Dual-functional luminescent Zn-MOF@MCHS nanocomposite for TNP detection and copper(II) adsorptive removal, Dual-functional luminescent Zn-MOF@MCHS nanocomposite for TNP detection and copper(II) adsorptive removal. Sep. Purif. Technol..

[119] Wu D., Du C., Ma L., Xiao R., Tan L., Qi W. (2025). Fluorescent sensitive detection of luteolin in Chrysanthemum tea using boric acid functionalized binuclear Zn-Eu-MOFs. Dye. Pigment..

[120] Wang H., Qian X., An X. (2023). Visual fluorescence detection of ciprofloxacin by Zn-metal-organic framework@nanocellulose transparent films based on aggregation-induced emission. Int. J. Biol. Macromol..

